# Understanding the impact of tuberous sclerosis complex: development and validation of the TSC-PROM

**DOI:** 10.1186/s12916-023-03012-4

**Published:** 2023-08-08

**Authors:** Annelieke R. Müller, Michiel A. J. Luijten, Lotte Haverman, Wendela L. de Ranitz-Greven, Peter Janssens, André B. Rietman, Leontine W. ten Hoopen, Laura C. G. de Graaff, Marie-Claire de Wit, Anna C. Jansen, Tanjala Gipson, Jamie K. Capal, Petrus J. de Vries, Agnies M. van Eeghen

**Affiliations:** 1 ‘s Heeren Loo, Amersfoort, The Netherlands; 2 Emma Center for Personalized Medicine, Department of Pediatrics, Amsterdam UMC Location University of Amsterdam, Amsterdam, The Netherlands; 3 Amsterdam Gastroenterology Endocrinology Metabolism, Amsterdam UMC, Amsterdam, The Netherlands; 4 Amsterdam Public Health Research Institute, Methodology and Mental Health and Personalized Medicine, Amsterdam, The Netherlands; 5 Department of Child and Adolescent Psychiatry & Psychosocial Care, Emma Children’s Hospital, Amsterdam UMC Location University of Amsterdam, Amsterdam, The Netherlands; 6 Amsterdam Reproduction & Development, Child Development, Amsterdam, The Netherlands; 7 Department of Internal Medicine, University Medical Center Utrecht, Utrecht, The Netherlands; 8 Department of Nephrology and Arterial Hypertension, Universitair Ziekenhuis Brussel (UZ Brussel), Vrije Universiteit Brussel, Brussels, Belgium; 9 Department of Child and Adolescent Psychiatry/Psychology and ENCORE Expertise Center, Erasmus Medical Center Sophia Children’s Hospital, Rotterdam, The Netherlands; 10 Erasmus School of Health Policy & Management, Erasmus University Rotterdam, Rotterdam, The Netherlands; 11 Center for Adults With Rare Genetic Syndromes, Division of Endocrinology, Department of Internal Medicine, Erasmus University Medical Center Rotterdam, Rotterdam, The Netherlands; 12 Department of Pediatric Neurology and ENCORE Expertise Center, Erasmus Medical Center Sophia Children’s Hospital, Erasmus University Medical Center Rotterdam, Rotterdam, The Netherlands; 13 Neurogenetics Research Group, Reproduction Genetics and Regenerative Medicine Research Cluster, Vrije Universiteit Brussel, Brussels, Belgium; 14 Pediatric Neurology Unit, Department of Pediatrics, Antwerp University Hospital; Translational Neurosciences, University of Antwerp, Antwerp, Belgium; 15 Department of Pediatrics, University of Tennessee Health Sciences Center, Memphis, TN USA; 16 Le Bonheur Children’s Hospital and Boling Center for Developmental Disabilities, Memphis, TN USA; 17 Department of Neurology, Cincinnati Children’s Hospital Medical Center, Cincinnati, OH USA; 18 Department of Neurology, University of North Carolina at Chapel Hill, Chapel Hill, NC USA; 19 Centre for Autism Research in Africa (CARA), Division of Child & Adolescent Psychiatry, University of Cape Town, Cape Town, South Africa

**Keywords:** Tuberous sclerosis complex, Patient-reported outcome measure, Functioning, Quality of life, Intellectual disability, Adults, Validity, Rare genetic disorder

## Abstract

**Background:**

Tuberous sclerosis complex (TSC) is a rare and complex genetic disorder, associated with tumor growth in various organ systems, epilepsy, and a range of neuropsychiatric manifestations including intellectual disability. With improving patient-centered care and targeted therapies, patient-reported outcome measures (PROMs) are needed to measure the impact of TSC manifestations on daily functioning. The aim of this study was to develop a TSC-specific PROM for adults that captures the impact of TSC on physical functions, mental functions, activity and participation, and the social support individuals with TSC receive, called the TSC-PROM.

**Methods:**

COSMIN methodology was used to develop a self-reported and proxy-reported version. Development and validation consisted of the following studies: PROM development, content validity, structural validity, internal consistency, and construct validity. The International Classification of Functioning and Disability was used as a framework. Content validity was examined by a multidisciplinary expert group and cognitive interview study. Structural and construct validity, and internal consistency were examined in a large cohort, using confirmatory factor analysis, hypotheses testing, and Cronbach’s alpha.

**Results:**

The study resulted in an 82-item self version and 75-item proxy version of the TSC-PROM with four subscales (physical functions 18 and 19 items, mental functions 37 and 28 items, activities and participation 13 and 14 items, social support 13 items, for self version and proxy version respectively). Sufficient results were found for structural validity with sufficient unidimensionality for each subscale. With regard to construct validity, 82% of the hypotheses were met for the self version and 59% for the proxy version. The PROM showed good internal consistency (Cronbach’s alpha 0.78–0.97).

**Conclusions:**

We developed a PROM for adults with TSC, named TSC-PROM, showing sufficient evidence for reliability and validity that can be used in clinical and research settings to systematically gain insight into their experiences. It is the first PROM in TSC that addresses the impact of specific TSC manifestations on functioning, providing a valuable, patient-centered addition to the current clinical outcomes.

**Supplementary Information:**

The online version contains supplementary material available at 10.1186/s12916-023-03012-4.

## Background

Tuberous sclerosis complex (TSC) is a rare autosomal dominant genetic disorder with a prevalence of 1 in 6000, caused by pathogenic variants in the *TSC1* or *TSC2* genes [1]. TSC is characterized by benign tumor growth in various organ systems, including the skin, kidneys, lungs, heart, and brain [2]. Epilepsy is a common feature of TSC and is often present in the first year of life (80%) [3]. In addition, TSC is associated with varying degrees of intellectual disabilities (ID) (50%) [4] and TSC-associated neuropsychiatric disorders (TAND) (90%) [5], which encompass psychiatric, behavioral, intellectual, neuropsychological, academic, and psychosocial manifestations [3, 4]. The severity of TSC manifestations can vary greatly but health perception and functioning are often severely impaired [6–9].

With improved healthcare, the largest population with TSC is now adult. Thus far, little is known of the burden and restrictions experienced by adults with TSC and the impact of TSC on functioning. As there is great variability in the severity of organ-specific involvement per life phase [2], adult care is often variable and fragmented, including gaps in care for TAND [5, 10–12]. Therefore, measuring the impact of various manifestations of TSC on functioning is both important and challenging and could improve care and allow monitoring over time. Moreover, if individuals with TSC have learning difficulties and mental health problems, they may have difficulties indicating their symptoms or healthcare needs, resulting in unknown and hence unmet healthcare needs. This could, in turn, lead to impaired functioning [10, 13, 14].

Various outcomes have been measured to assess disease severity in TSC research. Clinical or surrogate outcomes are often narrow in their focus, and it is unclear whether changes are relevant. For instance, although (severity of) epilepsy has been directly related to functioning [6, 15], reduction of seizure frequency does not always lead to improved functioning [16, 17]. In addition, what clinicians consider relevant is not identical to what individuals with TSC find important. The International Classification of Functioning and Disability (ICF) is a biopsychosocial model of disability based on an integration of the social and medical models of disability (World Health Organization 2001). The ICF conceptualizes a person’s level of functioning as a dynamic interaction between health conditions, environmental factors, and personal factors.

To get a better understanding of functioning and what is relevant to individuals, a patient-reported outcome measure (PROM) would allow an insight into perceived severity and impact. PROMs are questionnaires that measure how an individual experiences his or her own health [18–20]. They have become important for value-based healthcare and shared-decision making [21] and are increasingly used in practice and scientific research to quantify the severity and impact of the diseases on daily functioning from the perspective of the individual. PROMs enable periodical and quantitative evaluation of symptoms and functioning of the patient population. It can thus be used for monitoring and informing care, and as an outcome measure for trials [22].

Questionnaires commonly used in TSC trials, such as the Pediatric Quality of Life Inventory (PedsQL™ 4.0) [23] and Short-Form 36 (SF-36) [24], do not include disease-specific symptoms and may not be responsive enough for individuals with TSC [25, 26]. In addition, adults with TSC may or may not be able to self-report, and most existing questionnaires for adults are most commonly solely available as self-report. Adult proxy-report questionnaires are often unavailable for the domains of interest. It has been suggested that health problems in TSC are underestimated by excluding the more severely affected individuals, preventing them from early interventions [27–29]. Previous clinical trials that did not demonstrate significant clinical benefits based on parent-reported PROMs as primary outcome measures, such as the Aberrant Behavior Checklist – Irritability subscale [30], have been considered unsuccessful even when secondary outcome measures, such as visual analog scale ratings of parent-nominated problem behaviors or subscales validated for that specific patient population, indicated positive improvements [31]. This raises questions about whether the intervention was truly ineffective or whether the measurement instrument or mode of administration (proxy-report) was not responsive to therapy or suitable for the population being studied.

Especially now that disease-modifying and often long-term and expensive therapies are increasingly available, there is an urgent need for a TSC-specific PROM to measure effects of clinical parameters and treatment on disease-specific functioning, in both clinical and research settings. The use of a TSC-specific PROM in clinical trials can provide valuable evidence of the risks and benefits of treatments from a patient perspective which can inform regulatory approvals, clinical guidelines, and health policy, as it captures information that is relevant to the individual with TSC [32]. Therefore, the development of a reliable and valid instrument that measures domains and symptoms relevant to individuals with TSC is a top priority for patient organizations, researchers, and healthcare providers [5, 33, 34].

The aim of the current study was to develop and validate a TSC-specific PROM that captures the impact of TSC on physical and mental functions, activity and participation, and social support received by individuals with TSC, using the framework of the ICF (World Health Organization, 2001). The questionnaire is called TSC-PROM and consists of separate versions in English and Dutch for self-report and proxy-report, with the latter being the most suitable option to receive information about the possibly experienced issues for individuals who are unable to report on themselves.

## Methods

**Fig. 1:**
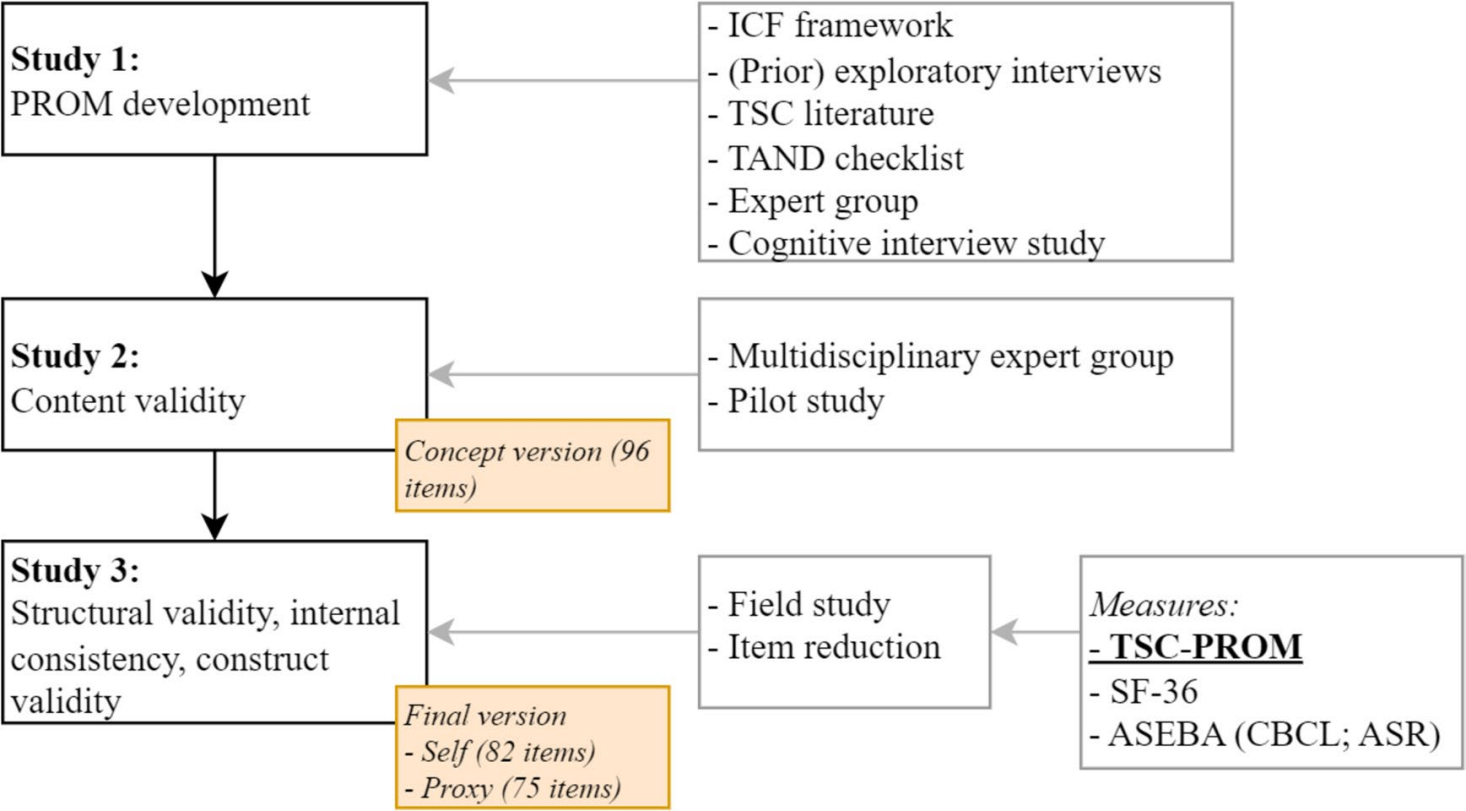
Flowchart of development and validation of the TSC-PROM, according to the standards from the COSMIN. ASEBA, Achenbach System of Empirically Based Assessment; ASR, Adult Self Report; CBCL, Child Behavior Checklist; ICF, International Classification of Functioning and Disability; PROM, patient-reported outcome measure; SF-36, Short-Form 36; TAND, TSC-associated neuropsychiatric disorders

Standards from the COnsensus-based Standards for the selection of health Measurement INstruments (COSMIN) were used to develop the questionnaire [33, 34]. Development and validation consisted of the following studies: (1) PROM development, (2) content validity, and (3) structural validity, internal consistency, and construct validity (Fig. 1).

### Study 1: PROM development

#### Construct and target population

The construct to be measured is the impact of TSC on physical functions, mental functions, activity and participation, and the social support individuals with TSC receive, using the framework of the ICF (World Health Organization 2001).

The purpose of the TSC-PROM is to evaluate and monitor the impact of TSC on functioning, serving as a tool to facilitate detection and discussion of healthcare needs relevant to individuals with TSC before or during a clinical visit. Two versions of the questionnaire were developed: a self-rated questionnaire for individuals with TSC without ID or a mild ID and a proxy-rated questionnaire for parents and caregivers of adults with TSC who could not complete the questionnaire themselves due to ID severity as assessed by primary caregivers or legal representatives.

The TSC-PROM was developed for all adults (18 years or older) diagnosed with TSC. To fill out the questionnaires, individuals with TSC, parents, or caregivers were English or Dutch-speaking.

#### Concept elicitation (relevance and comprehensiveness)

Relevant themes were identified by conducting interviews with adults with TSC and caregivers of adults [10]. The TSC-Associated Neuropsychiatric Disorders (TAND) checklist, Lifetime Version (TAND-L) [4], and TSC literature on adult manifestations were reviewed to identify additional themes [1, 10, 35–37]. The TAND checklist was specifically designed as a screening tool for neuropsychiatric manifestations of TSC, and validated, showing sufficient internal consistency and external validity [38]. Additionally, representatives of patient organizations were asked to identify additional themes. Expert meetings with an expert group representing various disciplines including neurology, psychiatry, psychology, endocrinology, nephrology, ID physician, methodological experts, and representatives of individuals with TSC were held to identify and assess the relevance of the themes until consensus was reached (AvE, AR, MdW, LdG, ET, PJ, PdV, LtH, JvdE).

After identifying relevant themes, the expert group categorized TSC-relevant themes by using the framework of the ICF [39]. The ICF delineates several domains, including the components *health condition*, *body functions and structure*, *activity, participation*, *environmental factors*, and *personal factors*. These components were used to classify the TSC-PROM subscales. The component *body functions and structure* was divided into the domains (1) *physical functions* and (2) *mental functions*. The ICF components *activity* and *participation* were combined into one domain (3) *activities and participation*. The fourth subdomain *social support* was composed of the ICF component *environmental factors*.

From the identified themes, simple and quantitative questions with response options using 4-point Likert scales were formulated by the expert team. Higher scores indicated better situations. The response options and recall period of the past month were chosen based on the expert opinion. Additionally, visual analog scales (VAS) were included per domain. We also included one question about their health-related quality of life (HRQoL). The preliminary test versions of the questionnaires for individuals with TSC and proxies were reviewed and refined by the expert group. Based on their expertise, topics were added, altered, or removed.

#### Translation

Two questionnaires were initially developed in the Dutch language in the Netherlands and Belgium. After professional expert translation into English using back and forward translation, a pilot was performed in the USA with English-speaking individuals with TSC and representatives (at least five self-rated and five proxy-rated) in order to broaden the population. The pilot concluded with a discussion with the expert group, and final revisions were made to the questionnaires with a final consensus.

#### Cognitive interview study (comprehensibility)

A cognitive interview study was performed in all participating languages (Dutch and English) to assess the comprehensibility of the questionnaire. Other than those who participated in the concept elicitation, individuals with TSC from the participating outpatient clinics were asked to provide qualitative feedback on the questionnaire. At least five participants were recruited per type of report (self or proxy) with a definite diagnosis of TSC [2] and a minimum age of 18 years for each participating country (the Netherlands, Belgium, and the USA), with an aimed minimum of 30 participants. We aimed to include participants with different ages, gender, and education to have a sample representing the target population. Individuals with TSC were excluded when an additional genetic disorder to TSC was diagnosed. The cognitive interview study included the “Think aloud” method [33, 40, 41] and the “Retrospective Verbal Probing” technique to assess comprehensibility of the instructions, all items, response options, and recall period [41].

### Study 2: Content validity

Face and content validity was examined by the multidisciplinary expert group and with the abovementioned participants from the cognitive interview study in the Netherlands, Belgium, and the USA; these countries were selected by convenience. Individuals with TSC from the participating outpatient clinics were asked to provide feedback on the digital questionnaire by completing a feedback form by using the “Retrospective Verbal Probing” technique [41] and a topic guide by interviewers who were trained specifically for the study. The feedback form consisted of questions on relevance, comprehensiveness, comprehensibility, and practical issues such as ease of use and lay-out. The TSC-PROM was considered feasible when time to complete was below 30 min. Comprehensibility was considered sufficient when at least 75% of the participants agreed on clarity of the instructions, items, formulations, response options, and sequence of items. Also, individuals with TSC were asked whether there were missing, redundant, or unclear items, as well as the most important TSC manifestations, to identify possible missing themes. Cognitive debriefing was performed to refine further and focus the items of the questionnaire and to gain insight into the instrument’s practical applicability. Group meetings and interviews were recorded and transcribed verbatim. Two researchers were involved in the analysis. The pilot phase concluded with a final discussion with the expert group about whether each item was relevant for the construct of interest and comprehensiveness of the TSC-PROM..

### Study 3: Structural validity, internal consistency, construct validity

#### Procedure

E-mails with an invitation link and login code granting access to the questionnaires were sent to participants after answering a question about whether the questionnaire will be filled out by either the individual with TSC themselves or a proxy when (assisted) self-report was not possible, as indicated by the primary caregiver, legal representative, or clinician. A proxy who declared to know the individual with TSC well, such as the legal representative or primary caregiver, was allowed to fill out the questionnaires.

#### Participants

To be eligible for participation in this study, adults (18 years or older) with a definite diagnosis of TSC, molecularly or clinically confirmed according to recent recommendations [1, 2], should be English or Dutch-speaking. Participants were recruited from the outpatient TSC clinics at the University Medical Center of Utrecht (Utrecht, the Netherlands), Erasmus Medical Center (Rotterdam, the Netherlands), the University Hospital of Brussels (Brussels, Belgium), Le Bonheur Children’s Hospital Memphis (Memphis, USA), and Cincinnati Children’s Hospital Medical Center (Cincinnati, USA). Additionally, participants were recruited via the Dutch (STSN), Belgian (beTSC), and US (TSC Alliance) patient organizations.

#### Measures

In addition to the TSC-PROM, the SF-36 [24], including a proxy-report [42], and scales assessing emotional and behavioral problems from the Achenbach System of Empirically Based Assessment (ASEBA) [43, 44], i.e., the Adult Self Report (ASR), the Child Behavior Checklist (CBCL)/1.5–5 and CBCL/6–18 were used for assessing construct validity. Individuals who were mentally competent to fill out the questionnaires themselves received the ASR. For individuals with TSC who could not complete the questionnaires, caregivers or representatives indicated whether the developmental age was below or above the age of 6 years old, guiding the distribution of either the CBCL/1.5–5 or CBCL6-18 version. Information on measurement properties of these comparator instruments is provided (see Additional file 1) [24, 27, 38, 42–62].

#### Item reduction

Item reduction was performed by selection based on frequency (at least 85% with response option “Not at all”), factor loadings, monotonicity, or local independence unless there was a clinical reason to include the item based on expert opinion. Additionally, items of the proxy version were reduced when frequency of the response options “Don’t know” and “Not applicable” was at least 30%.

#### Statistical analyses

Statistical analyses were performed using IBM SPSS Statistics 24 and R. Descriptive statistics were used to characterize demographics, clinical variables, and score distributions of the TSC-PROM. Domain scores were calculated as a percentage of the sum of the items within a domain. For the social support domain, the sum was divided by the number of items filled in other than “unknown” or “not applicable” times the number of response options to account for the “unknown” and “not applicable” response options. The TSC-PROM total score was the average of the domain percentages, excluding the social support domain. The social support domain was included as a scale to gather information on the type and quantity of the support someone is receiving, which is an important part of the PROM for the sake of completeness, as this could affect physical functions, mental functions, and activities and participation, but not directly a functioning component. A two-sided significance level of 5% was used.

Structural validity was assessed for the subscales (1) physical functions, (2) mental functions, and (3) activities and participation using a confirmatory factor analysis (CFA) (see Additional file 1).

With regard to internal consistency, Cronbach’s alpha was calculated for each TSC-PROM subscale, including the continuous HRQoL VAS. A Cronbach’s alpha between 0.70 and 0.95 was considered adequate [63].

Construct validity was examined by correlating the scores of the TSC-PROM with scores of other instruments that assess the same construct to be measured, also known as convergent validity. Regarding convergent validity, correlations were assessed between the TSC-PROM domain scores and the SF-36 physical component score, mental component score, and the total scores of the ASR, CBCL/1.5–5 or CBCL/6–18. Construct validity was considered sufficient if 75% of the hypotheses were met (see Additional file 1). To assess discriminative validity, analyses were performed using group dichotomization or categorization. A priori hypotheses were defined including (1) individuals with TSC2 pathogenic variants will show lower TSC-PROM scores on the physical domain, mental domain, and TSC-PROM HRQoL VAS compared to individuals with a TSC1 pathogenic variant [60–62]; (2) individuals who reported a drastic life event in the past year will show a lower score on the mental functions domain; (3) individuals with a higher number of involved organ systems will show lower scores on the HRQoL VAS [27]; and (4) individuals with the presence of psychiatric diagnoses will show lower TSC-PROM scores on the mental functions domain, activities and participation domain, and HRQoL VAS [27] (see Additional file 1).

#### Data management

The questionnaires were digitally distributed using LimeSurvey [64]. As the survey did not allow for missing data, no specific missing item analysis was necessary. Only the principal investigator (AvE) and researcher (AM) had access to the code for each participant that was solely accessible in the secure network environment of the Erasmus Medical Center. Data were stored in LimeSurvey and exported to R for statistical analyses.

## Results

### Study 1: PROM development

#### Concept elicitation

Concept elicitation resulted in a draft version of the TSC-PROM for both self and proxy-report (73 items from prior exploratory interviews [10]; supplementary 6 items from TSC literature on adult manifestations; 9 items from the TAND checklist [4]; 11 items from the expert group). After an expert meeting, some items were divided into separate items or combined, resulting in a total of 96 items (24 items within the physical functions domain, 43 items within the mental functions domain, 19 items within the activities and participation domain, and 9 items within the social support domain).

The TSC-PROM starts with 15 questions on demographic and clinical information, including clinical and sociodemographic information (age, sex, nationality, age of TSC diagnosis, genetic testing, organs involved, use of medication, epilepsy, level of functioning, educational level, other diagnoses or health conditions, life events). Visual analog scales from 0 to 100 were included on physical functions, mental functions, the ability to perform daily activities, and satisfaction with social support, and a HRQoL VAS was included. During item development, response options were defined using Likert scales with higher scores indicating overall less impairment. To illustrate, “a lot” (1), “somewhat” (2), “a little” (3), and “not at all” (4) were response options for items on the physical functions and mental functions domain, such as “During the past month I was bothered by [e.g. difficulty sleeping, skin abnormalities, seizures]” and “During the past month I [e.g. experienced restlessness/insecurity/difficulty in meeting new people, felt anxious, had mood swings, I worried about tumor growth/my financial independence].” Response options for the activities and participation domain included “always” (1), “often” (2), “sometimes” (3), and “never” (4) with items such as “During the past month I was limited in [e.g. learning something new, getting along with people I know well, participating in sport/physical exercise].” Response options for the social support domain included “not at all” (1), “a little” (2), “mostly” (3), and “completely” (4), “not applicable" with items such as “In the past month I was satisfied with [e.g. the support I received from my family/partner/mental healthcare professionals, how my medication is working].”

#### Cognitive interview study

We recruited eleven participants (five self-rated and six proxy-rated) in the Netherlands, ten in Belgium (five self-rated and five proxy-rated), and ten in the USA (five self-rated and five proxy-rated), with a definite diagnosis of TSC [2] and an average age of 34.43 years (range 18–65 years). The questionnaires were completed for eighteen female participants and thirteen male participants. The level of ID differed from fourteen without ID, six with a mild ID, five with a moderate ID, and six with a severe ID. Based on the feedback received during the interview study, the following adjustments were made:

- An introduction was added for each domain to emphasize the subjective experience of possible complaints and how to deal with structurally present complaints.
- Some questions (mainly regarding the demographic and clinical information) were adjusted and reformulated to abate any confusion and redundant information, and to specify some manifestations, such as frequency of seizures and life events.
- Some items were formulated reversely (e.g., “I like meeting other people”), while the majority was about the burden and complaints, causing confusion. These questions were reformulated.

### Study 2: Content validity

Content and face validity of the questionnaire were ensured by involving TSC experts in the field, including individuals with TSC and representatives in focus group interviews and the expert multidisciplinary team. In this way, the instrument’s content validity was verified by all major stakeholders. We recruited eleven participants (five self-rated and six proxy-rated) in the Netherlands, ten in Belgium (five self-rated and five proxy-rated), and ten participants in the USA (five self-rated and five proxy-rated).

#### Feasibility

It took participants 16.53 (± 5.00, range 10–30) minutes to complete the questionnaire. Participants preferred a digital version and the lay-out was assessed as clear by 85.7% of the participants and 14.3% somewhat agreed.

#### Comprehensibility

Eighty-one percent of the participants found the instructions, the items, and the formulations clear, and 19% somewhat agreed. One participant indicated difficulties when complaints are always present. Small suggestions were made for clarification. 85.7% of the examples provided were clear and understandable. 66.7% indicated clear response options. Feedback included lack of the response option “not applicable” in the self-report version and difficulty to estimate the applicability of “not applicable” or “do not know” for the proxy-report. 85.7% of the participants agreed on the sequence of items.

#### Relevance and comprehensiveness

Participants did not indicate other items or complaints and agreed on comprehensiveness, completeness, and relevance. All relevant questions were included, although not all questions were applicable to the different levels of functioning.

### Study 3: Structural validity, internal validity, construct validity

E-mails with access to the questionnaires were sent to 210 participants, with a response rate of 78%. In total, 163 participants completed the TSC-PROM, of whom 114 participants filled in the complete questionnaire battery (85% of self-reporting participants and 46% of proxy-reporting participants). Six and thirteen self-reporting participants and 27 and 36 proxy-reporting participants did not fill out the SF-36 and ASEBA questionnaires, respectively. The sociodemographic and demographic and clinical characteristics are presented in Table 1. Seven participants reported other nationalities, including Canadian, Australian, British, Spanish, and Finnish.

**Table 1:** Sociodemographic and clinical characteristics of participants

Sociodemographic and clinical characteristics	Self (*n* = 85)		Proxy (*n* = 78)	
		**%**		**%**
*Age (years)*	43.3 (range 18–73)		34.8 (range 18–66)	
*Sex*				
*Female*	49	57.6	33	42.3
*Male*	35	41.2	45	57.7
*Other*	1	1.2	0	0.0
*Nationality*				
*American*	14	16.5	7	9.0
*Dutch*	61	71.8	63	80.8
*Belgium*	6	7.1	5	6.4
*Other (Canadian, Australian, British, Spanish, Finnish)*	4	4.7	3	3.8
*Age diagnosis TSC (years)*	20.7 (range 0–59)		4.2 (range 0–35)	
*Genetic cause*^a^				
*TSC1 pathogenic variant*	15	17.6	6	7.7
*TSC2 pathogenic variant*	23	27.1	27	34.6
*No pathogenic variant identified*	4	4.7	5	6.4
*Variant of unknown significance*	3	3.5	3	3.8
*Result unknown*	18	21.2	25	32.1
*Not genetically tested or unknown*	22	25.9	12	15.4
*Organs showing symptoms of TSC (e.g., tubers, tumors, pigment changes)*				
*None*	1	1.2	0	0.0
*Brain*	60	70.6	72	92.3
*Skin*	71	83.5	72	92.3
*Kidneys*	73	85.9	72	92.3
*Lungs*	29	34.1	11	14.1
*Eyes*	20	23.5	20	25.6
*Heart*	21	24.7	32	41.0
*Mouth*	18	21.2	13	16.7
*Other (liver, nails, ovaria, pancreas, uterus, teeth, breast, colon, adrenal, intestines, rectum, ears, nose)*	16	18.8	18	23.1
*Use of medication*	58	68.2	77	98.7
*Anti-seizure drugs*	24	28.2	63	80.8
*mTOR inhibitors*	29	34.1	34	43.6
*Anti-hypertensive drugs*	11	12.9	14	17.9
*Other (e.g., antidepressants, antipsychotics, antihistamines, proton pump inhibitors)*	26	30.6	46	59.0
*Epilepsy (current or past)*	33	38.8	73	93.6
*Age first seizure*	6.5 (range 0–58)		1.3 (range 0–27)	
*Level of intellectual functioning*				
*Normal intellectual ability*	64	75.3	1	1.3
*Normal intellectual ability with specific learning disability (dyscalculia, dyslexia)*	12	14.1	0	0.0
*Mild or moderate ID*	9	10.6	31	39.7
*Severe or profound ID*	0	0.0	46	59.0
*Living situation*				
*Alone*	16	18.8	8	10.3
*With partner (and/or family)*	49	57.6	2	2.6
*With my parents (and siblings)*	14	16.5	24	30.8
*With roommates, friends or others*	6	7.1	42	53.8
*Support (living)*				
*Without assistance*	71	83.5	0	0.0
*Ambulatory professional support (no 24-h care)*	8	9.4	13	16.7
*Ambulatory professional support (with 24-h care)*	6	7.1	65	83.3
*Other diagnoses*^a^				
*Autism spectrum disorders (ASD)*	6	7.1	39	50.0
*Attention deficit (hyperactivity) disorders (AD(H)D)*	3	3.5	8	10.3
*Obsessive–compulsive disorders (OCD)*	3	3.5	5	6.4
*Anxiety disorder*	10	11.8	8	10.3
*Depressive disorder*	14	16.5	7	9.0
*Psychotic disorder*	0	0.0	6	7.7
*Relation to the individual*				
*Father*			20	25.6
*Mother*			41	52.6
*Sibling*			6	10.3
*Caretaker*			2	6.4
*Other*			5	5.1

^a^As reported by the primary caregiver or legal representative who completed the questionnaires

#### Item reduction

The TSC-PROM consisting of 96 items and the five visual analog scales was subjected to item reduction by applying the criteria defined in the method section, unless there was a clinical reason to include based on expert opinion. Two items were included based on expert opinion, namely the burden of seizures and kidneys over the past month. After item reduction, the self version contained 82 items and the proxy version 75 items (physical functions domain 18 items and 19 items with an additional item on side effects, mental functions domain 37 items and 28 items, activities and participation domain 13 items and 14 items, social support 13 items, for self-report and proxy-report respectively) (see Additional file 1). Items in the proxy version that relied on internal perception or were difficult to estimate as a proxy were removed, such as “*the individual felt lonely*.”

#### Structural validity

The mental functions and activities and participation self-report scales displayed sufficient unidimensionality and monotonicity according to the predefined criteria (Table 2). The physical self-report scale and the proxy-report scales did not or only partially satisfy the unidimensionality or monotonicity assumption. Some items within the self-report scale displayed local dependence (residual correlation > 0.20; physical functions domain: 4.90%, mental functions domain: 5.03%, activities and participation domain: 5.77%). Within the proxy-report scale, some items showed local dependence (physical functions domain: 8.77%, mental functions domain: 10.46%, activities and participation domain: 9.34%).

**Table 2:** Unidimensionality and monotonicity of the self and proxy version of the TSC-PROM

	**Self**			**Proxy**		
	*Physical functions*	*Mental functions*	*Activities and participation*	*Physical functions*	*Mental functions*	*Activities and participation*
***Unidimensionality***						
*CFA*						
CFI	0.90	**0.97**	**0.97**	0.88	0.90	**0.96**
TLI	0.89	**0.97**	**0.97**	0.86	0.90	**0.95**
RMSEA	**0.07**	**0.06**	0.11	**0.08**	**0.08**	0.12
*Bi-factor model*						
*ω*h	0.59	0.72	0.75	0.53	0.67	0.72
ECV	0.53	**0.60**	**0.64**	0.40	0.53	0.58
***Monotonicity***						
*H*i	> 0.10	** > 0.31**	** > 0.50**	> 0.02	> 0.06	> 0.21
H	0.30	**0.518**	**0.62**	0.27	0.37	0.49

Values in bold indicate acceptable fit. Predefined criteria are provided (see Additional file 1)

*CFA* Confirmatory factor analysis, *CFI* Comparative fit index, *ECV* Explained common variance, *RMSEA* Root mean square error of approximation, *TLI* Tucker-Lewis index

#### Internal consistency reliability

For the self-report, the corrected item-total correlations ranged from 0.00 to 0.62 (physical functions domain), 0.39 to 0.82 (mental functions domain), and 0.56 to 0.84 (activities and participation domain). For the proxy-report, it ranged from 0.00 to 0.72 (physical functions domain), 0.08 to 0.84 (mental functions domain), and 0.23 to 0.82 (activities and participation domain). Cronbach’s alpha value of the total TSC-PROM score was 0.819 and 0.775 for the self and proxy-report, respectively, which met the threshold criterion range of 0.70–0.95. Cronbach’s alpha of each subscale ranged from 0.81 to 0.97 (Table 3).

**Table 3:** Item-total correlations and internal consistency reliability of the TSC-PROM

	**Item-total correlations**		**Cronbach’s alpha**	
	*Self-report*	*Proxy-report*	*Self-report*	*Proxy-report*
Total TSC-PROM			0.819	0.775
Physical functions domain	0.00–0.62	0.00–0.72	0.806	0.820
Mental functions domain	0.39–0.82	0.08–0.84	0.967	0.924
Activities and participation domain	0.56–0.84	0.23–0.82	0.938	0.906

#### Construct validity

All hypotheses regarding construct validity were met for both the self version and proxy version (Table 4).

**Table 4:** Predefined hypotheses and results regarding construct validity of the TSC-PROM self and proxy version

**Predefined hypotheses**	**Results**			
	*Self (n* = *85)*		*Proxy (n* = *78)*	
Moderately strong correlation between TSC-PROM physical functions domain and SF-36 physical component score	Moderately strong	*r* = 0.60; *p* < 0.001	Moderately strong	*r* = 0.55, *p* < 0.001
Moderately strong correlation between TSC-PROM mental functions domain and SF-36 mental component score	Strong	*r* = 0.83, *p* < 0.001	Moderately strong	*r* = 0.53, *p* < 0.001
Moderately strong correlation between TSC-PROM mental functions domain and total ASR or CBCL scores	Strong	*r* = 0.87, *p* < 0.001	Moderately strong	*r* = 0.61, *p* < 0.001
Weak to moderately strong correlations between TSC-PROM domain score and TSC-PROM HRQoL VAS score				
Physical functions domain	Moderately strong	*r* = 0.59, *p* < 0.001	Weak	*r* = 0.44, *p* < 0.001
Mental functions domain	Moderately strong	*r* = 0.55, *p* < 0.001	Weak	*r* = 0.39, *p* < 0.001
Activities and participation domain	Moderately strong	*r* = 0.64, *p* < 0.001	Weak	*r* = 0.35, *p* = 0.003
Moderately strong correlations between TSC-PROM HRQoL VAS score and TSC-PROM VAS domain scores				
Physical functions domain	Moderately strong	*r* = 0.67, *p* < 0.001	Moderately strong	*r* = 0.57, *p* < 0.001
Mental functions domain	Moderately strong	*r* = 0.65, *p* < 0.001	Moderately strong	*r* = 0.61, *p* < 0.001
Activities and participation domain	Moderately strong	*r* = 0.62, *p* < 0.001	Moderately strong	*r* = 0.59, *p* < 0.001

Weak (*r* > 0.3), moderately strong (*r* > 0.5), and strong (*r* > 0.7) correlations

#### Discriminative validity

In the self-report, no significant differences were found between individuals with *TSC1* and *TSC2* pathogenic variants. Furthermore, individuals with TSC who experienced a life event showed a lower score on the TSC-PROM mental functions domain (*p* = 0.018, *r* =  − 0.26), patients with a higher number of organ manifestations showed a lower HRQoL VAS score (*p* = 0.021, *r* =  − 0.25), and individuals with TSC with the presence of psychiatric diagnoses showed lower TSC-PROM scores on the mental functions domain (*p* < 0.001, *r* =  − 0.47), the activities and participation domain (*p* < 0.001, *r* =  − 0.40), and HRQoL VAS score (*p* = 0.040, *r* =  − 0.22). In the proxy-report, individuals with *TSC2* pathogenic variants showed a lower TSC-PROM score on the mental functions domain compared to individuals with a *TSC1* pathogenic variant (*p* = 0.012, Cohen’s *D* = 0.69). No significant differences were found with regard to the experience of a life event, the number of organ manifestations, and the presence of psychiatric diagnoses.

For the self-report, all of the hypotheses were met, except for the hypotheses regarding the *TSC1* and *TSC2* pathogenic variants, resulting in a total of 63% of hypotheses met. For the proxy-report, one out of eight hypotheses was met (13%), which was the hypothesis regarding the effect of *TSC1* and *TSC2* pathogenic variants on the mental functions domain.

## Discussion

The TSC-PROM is the first TSC-specific outcome measure comprehensively addressing all relevant aspects of the ICF model for adults with TSC. It is developed and validated according to the gold standard COSMIN, with versions for proxies of individuals with TSC who are unable to use it themselves (see Additional file 2 and 3). The TSC-PROM may be used in both research and clinical settings to assess physical and mental functions, activity and participation, and social support individuals with TSC receive. To date, the TSC-PROM is available in English and Dutch, but translation into other languages and an accessible digitalized version will allow broader evaluation and application of this TSC-specific PROM.

### Psychometrics

Psychometric evaluation shows that the TSC-PROM has sufficient validity and reliability to serve as an instrument to systematically gain insight into the impact of TSC on physical functions, mental functions, and activity and participation and the social support individuals with TSC receive and provides a vital addition to current clinical outcomes.

The most important part of the development of the TSC-PROM is content validity [65] which was ensured and verified by all major stakeholders, including individuals with TSC and a broad multidisciplinary team of TSC experts. Some adjustments were made based on the feedback received during the cognitive interview study in the participating countries, and feasibility, comprehensibility, relevance, and comprehensiveness were demonstrated. However, cross-cultural validity has not yet been examined, and cultural adaptations may be necessary when using the TSC-PROM in other countries and languages. Satisfactory results were demonstrated on internal consistency and structural validity. Unidimensionality was satisfied, but there was some overlap between items indicated by local dependencies. This may be explained by the fact that items were divided into clusters with overlap in content of symptoms which often co-exist. Satisfactory results were also demonstrated on construct validity, although not all hypotheses with regard to discriminative validity were met, in particular for the proxy version. These results may reflect the heterogeneity of the TSC population and indicate that function for individuals with TSC is difficult to determine by proxy-reports [42]. Furthermore, higher scores of the TSC-PROM indicating better functioning were observed for self-ratings compared to proxy-ratings (*p* < 0.001, *r* =  − 0.50), perhaps because the proxy-ratings concern individuals who are more affected by the neurological manifestations of TSC or due to bias of the rating as in other studies proxy-raters often seem to assess functioning as worse [66–68].

### Recommendations for use in the care setting

The TSC-PROM can provide quantitative evaluation of the severity and impact of TSC on various health domains and daily functioning from the patient’s perspective. As such, it might be used for monitoring and informing care. The instrument might also serve as a tool to facilitate detection of healthcare needs before or during a clinical visit. Although it is an elaborate questionnaire and it might take some time to complete, it consists of all relevant items. However, not all items or domains are always applicable to individuals with TSC due to the heterogeneity and treatment goals. Therefore, a subdomain could be used as well rather than the whole instrument, although it might still be valuable to use all domains in order to not forget about possible manifestations. It ensures an effective follow-up and timely referral to appropriate care providers. Until now, assessments of disease severity using clinical rating scales such as the clinical global impression scale omitted patient perspectives about issues of relevance to their health. Additionally, it has been pointed out that perception of the individuals’ functioning by clinicians and individuals themselves differ [69, 70]. Using the TSC-PROM may improve communication between the individual and clinician and treatment outcomes and facilitate shared-decision making, resulting in increased satisfaction with care.

### Recommendations for use in research

The TSC-PROM can bridge the gap between care and interventional research. It can be used as an outcome measure to gain insight into patients’ perspective on physical functions, mental functions, activities and participation, and the social support individuals with TSC receive, in observational, epidemiological, and longitudinal studies and in interventional trials. It can also relate therapeutic or biomarker findings to self-evaluated functioning. This is important for evaluating novel treatments such as anti-seizure medication, mTOR inhibitors, cannabidiol treatments, and eventually more (expensive) targeted therapies such as gene or RNA modification [2, 71]. Although a TAND-specific outcome measure is under development [72], the assessment of all relevant health domains in individuals with TSC has been hampered by the lack of a TSC-specific measure [9], comparable to several other rare diseases for which disease-specific outcome measures have eventually been developed [28, 73–76].

Thus far, generic instruments have been used with the advantage of allowing comparison between different disease (sub)groups. However, these PROMs often do not include all relevant domains of functioning in TSC or proxy versions for adults are not available [10, 17]. As a result, multiple tools have been used in single trials to measure the full impact. As the TSC-PROM addresses all domains of the ICF framework relevant to individuals with TSC while displaying convergent validity to existing generic instruments (SF-36, ASR, CBCL), it may better capture all important manifestations and aspects that impact the functioning of individuals with TSC than existing instruments.

### Strengths, limitations, and future directions

The TSC-PROM provides an innovative tool to measure what is relevant to individuals with TSC, taking into account the complexity and heterogeneity of the clinical picture of TSC. It has been developed together with individuals with TSC and according to the gold standard COSMIN, providing high relevancy and good quality. It might serve as an example for future work for heterogeneous and complex disorders where existing instruments are unavailable for proxy-report and the domains of interest.

However, limitations of this study are the sample size and representation of a limited number of countries and languages, as there will be differences between countries and cultures regarding healthcare systems. According to COSMIN criteria, a sample size between 50 and 100 per age group is regarded a good sample size for establishing internal consistency and reliability in a PROM [34]. We aimed for a representative sample size of 200 participants, but a part of the participants did not complete the questionnaire battery. The majority of participants were from the Netherlands, although Belgium, American, Canadian, British, Spanish, and Finnish nationalities were included as well, as we recruited in the Netherlands, Belgium, and the USA without restrictions on nationality. In this study, we started to develop a Dutch and English instrument which was tested in the three participating countries. We have not yet examined the applicability for other countries, neither whether cultural adaptations are needed. Next, the TSC-PROM should be translated into other languages such that all individuals with TSC could benefit regardless of their language, country or culture, ensuring inclusivity.

Future interventional studies should evaluate responsiveness to change, test–retest validity and cross-cultural validity of the TSC-PROM and elaborate on discrepancies in functioning between self-reports and proxy-reports in which both the self and proxy versions are completed for one individual. Also, a shortened version of the TSC-PROM or more advanced psychometric methods such as item response theory (IRT-)based instruments might be developed for individuals with mild ID [77]. Ideally, a generic measure should be developed applicable to all rare genetic neurodevelopmental disorders with appropriate versions for different levels of ID with different symptom checklists to cover relevant disease-specific aspects, as it is not feasible and desirable to have disorder-specific PROMs for all these thousands of disorders.

## Conclusions

The TSC-PROM is the first TSC-specific outcome measure for adults with TSC, which has been developed using the ICF structure covering all relevant aspects of physical functions, mental functions, activities and participation, and social support and with input from individuals with TSC, caregivers, clinicians, as well as literature review and psychometric testing. It appears to have adequate to good psychometric properties of acceptability, reliability, and validity. This TSC-specific PROM provides a unique tool to systematically gain insight into the individuals’ experiences and monitor trial and therapy outcome, taking into account the complexity and heterogeneity of the clinical picture of TSC, and empowering TSC clinicians and researchers in the optimal care for adults with TSC.

### Supplementary Information

**Additional file 1.** Supplementary information with regard to methods and results. This file includes additional information with regard to measures, statistical analyses, predefined criteria, hypotheses, and item reduction with reasons for exclusion.

**Additional file 2.** TSC-PROM self and proxy version (English). The English self-report and proxy-report versions of the TSC-PROM.

**Additional file 3.** TSC-PROM self and proxy version (Dutch). The Dutch self-report and proxy-report versions of the TSC-PROM.

## Data Availability

The datasets generated and analyzed during the current study are not publicly available due to privacy reasons and sensitive information, but are available from the corresponding author on reasonable request.

## Abbreviations

AD(H)D: Attention deficit (hyperactivity) disorder
ASD: Autism spectrum disorder
ASEBA: Achenbach System of Empirically Based Assessment
ASR: Adult Self Report
BeTSC: Belgium patient organization for TSC
CBCL: Child Behavior Checklist
CFA: Confirmatory factor analysis
CFI: Comparative fit index
COSMIN: COnsensus-based Standards for the selection of health Measurement INstruments
ECV: Explained common variance
HRQoL: Health-related quality of life
ICF: International Classification of Functioning and Disability
ID: Intellectual disability
mTOR: Mammalian target of rapamycin
OCD: Obsessive-compulsive disorder
PedsQL: Pediatric Quality of Life Inventory
PROM: Patient-reported outcome measure
RMSEA: Root mean square error of approximation
RNA: Ribonucleic acid
SF-36: Short-Form 36
STSN: Dutch patient organization for TSC (Stichting Tubereuze Sclerosis Nederland)
TAND: TSC-associated neuropsychiatric disorders
TAND-L: TSC-Associated Neuropsychiatric Disorders (TAND) checklist, Lifetime Version
TLI: Tucker-Lewis index
TSC Alliance: Internationally recognized nonprofit for people with TSC
TSC: Tuberous sclerosis complex
VAS: Visual analog scale
