# Correction: Understanding the impact of tuberous sclerosis complex: development and validation of the TSC-PROM

**DOI:** 10.1186/s12916-023-03092-2

**Published:** 2023-10-23

**Authors:** Annelieke R. Müller, Michiel A. J. Luijten, Lotte Haverman, Wendela L. de Ranitz-Greven, Peter Janssens, André B. Rietman, Leontine W.ten Hoopen, Laura C. G. de Graaff, Marie-Claire de Wit, Anna C. Jansen, Tanjala Gipson, Jamie K. Capal, Petrus J. de Vries, Agnies M. van Eeghen

**Affiliations:** 1 ‘S Heeren Loo, Amersfoort, The Netherlands; 2 Emma Center for Personalized Medicine, Department of Pediatrics, Amsterdam UMC Location University of Amsterdam, Amsterdam, The Netherlands; 3 Amsterdam Gastroenterology Endocrinology Metabolism, Amsterdam UMC, Amsterdam, The Netherlands; 4 Amsterdam Public Health Research Institute, Methodology and Mental Health and Personalized Medicine, Amsterdam, The Netherlands; 5 Department of Child and Adolescent Psychiatry & Psychosocial Care, Emma Children’s Hospital, Amsterdam UMC Location University of Amsterdam, Amsterdam, The Netherlands; 6 Amsterdam Reproduction & Development, Child Development, Amsterdam, The Netherlands; 7 Department of Internal Medicine, University Medical Center Utrecht, Utrecht, The Netherlands; 8 Department of Nephrology and Arterial Hypertension, Universitair Ziekenhuis Brussel (UZ Brussel), Vrije Universiteit Brussel, Brussels, Belgium; 9 Department of Child and Adolescent Psychiatry/Psychology and ENCORE Expertise Center, Erasmus Medical Center Sophia Children’s Hospital, Rotterdam, The Netherlands; 10 Erasmus School of Health Policy & Management, Erasmus University Rotterdam, Rotterdam, The Netherlands; 11 Center for Adults With Rare Genetic Syndromes, Division of Endocrinology, Department of Internal Medicine, Erasmus University Medical Center Rotterdam, Rotterdam, The Netherlands; 12 Department of Pediatric Neurology and ENCORE Expertise Center, Erasmus Medical Center Sophia Children’s Hospital, Erasmus University Medical Center Rotterdam, Rotterdam, The Netherlands; 13 Neurogenetics Research Group, Reproduction Genetics and Regenerative Medicine Research Cluster, Vrije Universiteit Brussel, Brussels, Belgium; 14 Pediatric Neurology Unit, Department of Pediatrics, Antwerp University Hospital, Translational Neurosciences, University of Antwerp, Antwerp, Belgium; 15 Department of Pediatrics, University of Tennessee Health Sciences Center, Memphis, TN USA; 16 Le Bonheur Children’s Hospital and Boling Center for Developmental Disabilities, Memphis, TN USA; 17 Department of Neurology, Cincinnati Children’s Hospital Medical Center, Cincinnati, OH USA; 18 Department of Neurology, University of North Carolina at Chapel Hill, Chapel Hill, NC USA; 19 Centre for Autism Research in Africa (CARA), Division of Child & Adolescent Psychiatry, University of Cape Town, Cape Town, South Africa

**Correction: BMC Med 21, 298 (2023)**

**https://doi.org/10.1186/s12916-023-03012-4**

The original article [1] contained typographical errors in Additional Files 2 and 3 which have since been amended.

### Supplementary Information

**Additional file 2.**

**Additional file 3.**
