# Supplementary material for: Understanding the impact of tuberous sclerosis complex: development and validation of the TSC-PROM
Source: BMC Med. 2023 Aug 8;21:298. doi: 10.1186/s12916-023-03012-4 (PMC10408092; doi:10.1186/s12916-023-03012-4)
Supplement: Supplementary file 1 — Additional file 1. Supplementary information with regard to methods and results. This file includes additional information with regard to measures, statistical analyses, predefined criteria, hypotheses, and item reduction with reasons for exclusion. [file 12916_2023_3012_MOESM1_ESM.docx]

**Additional file 1**

**Methods**

*Measures*

The TAND checklist was used to identify additional items for the physical functions domain. It was specifically designed by a project team member as a screening tool for neuropsychiatric manifestations of TSC (38). It addresses developmental milestones, level of functioning, behavioral concerns, psychiatric disorders, intellectual ability, academic skills, psychosocial functioning, and ratings of the impact of the neuropsychiatric symptoms. The TAND checklist has been validated, showing good internal consistency and external validity (38,45).

For construct validity, the Short-Form-36 Health Survey (SF-36) (24) including a proxy-report, and scales assessing emotional and behavioural problems from the Achenbach System of Empirically Based Assessment (ASEBA) (43,44), i.e., the Adult Self Report (ASR), the Child Behavior Checklist (CBCL)/1.5-5 and CBCL/6-18 were used.

The SF-36 is a generic measure with 36 items, organized into eight multi-item scales assessing physical functioning, bodily pain, role limitation due to physical health problems, role limitation due to personal or emotional problems, emotional well-being, social functioning, energy/fatigue, and general health perceptions (46). A proxy version of the SF-36 was available from a previous study (42). Summary component scores for physical health (PCS) and mental health (MCS) were calculated, ranging from 0-100, with higher scores indicating better health status. The SF-36 has been commonly used in TSC research and showed evidence of validity and reliability as a measure of HRQoL in parents of children with mental illness (47-49).

The Adult Self Report (ASR) and the Child Behavior Checklist (CBCL) are part of the ASEBA questionnaires, assessing competencies (activities, social, school, and total), emotional state, and behavioural problems of children and adolescents (43,44,50). Total scores were used. The ASR and CBCL have been considered valid and reliable instruments and commonly used in TSC research (51-54).

*Statistical analyses*Structural validity, which is the degree to which the scores of the TSC-PROM are an adequate reflection of the dimensionality of the construct to be measured, was assessed for the subscales 1) physical functions, 2) mental functions, and 3) activities and participation. A confirmatory factor analysis (CFA) for each subscale with weighted least square mean- and variance-adjusted (WLSMV) estimator was performed to assess unidimensionality using the R-package “lavaan (v0.6-3)” (55,56). We used the following criteria for an acceptable CFA fit: Scaled Comparative Fit Index (CFI) and Tucker-Lewis Index (TLI) values>0.95, a standardized root mean square residual (SRMR) value <0.10, and a root mean square error of approximation (RMSEA) value <0.08 (57). If CFA fit did not meet the criteria (56), a bi-factor model was fit to assess whether unidimensionality was sufficient by assessing if the hierarchical omega (ωh) was >0.80 and the explained common variance (ECV) >0.60 (58). Local independence was assessed by the residual correlation in the CFA model. An item pair was considered to be locally independent if the residual correlation was <0.20 (56). Monotonicity was considered sufficient when the item H values of all items were ≥0.30 and the H value of the entire scale was ≥ 0.50, using Mokken scaling (59).

With regard to internal consistency, which refers to the degree of interrelatedness between items, Cronbach’s alpha was calculated for each TSC-PROM subscale including the continuous HRQoL VAS. A Cronbach’s alpha between 0.70 and 0.95 was considered adequate.

Construct validity, which refers to the assessment of the construct we aimed to assess, was examined by correlating the scores of the TSC-PROM with scores of other instruments that assess the same construct to be measured. Convergent validity is a subtype of construct validity and refers to the degree to which measures that theoretically should be related are in fact related. Regarding convergent validity, correlations were assessed between the TSC-PROM domain scores and the SF-36 physical component score, mental component score and the total scores of the ASR, CBCL/1.5-5 or CBCL/6-18. Assumptions were tested, including normality, linearity, homoscedasticity, and presence of significant outliers. A moderately strong correlation (Pearson’s or Spearman’s r > 0.5) was expected between 1) the TSC-PROM physical functions domain score and the SF-36 physical component score, 2) the TSC-PROM mental functions domain score and the SF-36 mental component score, and 3) the TSC-PROM mental functions domain score and the total ASR score or CBCL scores. Also, it was expected that TSC-PROM domains are associated with TSC-PROM VAS scores (weak correlations). Construct validity was considered sufficient if 75% of the hypotheses were met.

Another subtype of construct validity is discriminative validity, which refers to the degree that measures of constructs that theoretically should not be highly related to each other are in fact not found to be highly correlated to each other. To assess discriminative validity, analyses were performed using group dichotomization or categorization. A priori hypotheses were defined including 1) patients with TSC2 mutations will show lower TSC-PROM scores on the physical domain, mental domain, and TSC-PROM HRQoL VAS compared to patients with a TSC1 mutation (60-62), 2) patients who reported a drastic life event in the past year will show a lower score on the mental functions domain, 3) patients with a higher number of involved organ systems will show lower scores on the HRQoL VAS (27), and 4) patients with the presence of psychiatric diagnoses will show lower TSC-PROM scores on the mental functions domain, activities and participation domain, and HRQoL VAS (27). Assumptions were tested, including normality and homogeneity of variances. Independent samples t-tests or Mann-Whitney U-tests were performed. Effect sizes were calculated via r = z/√N.

**Results**

Item reduction of the TSC-PROM self-report and proxy-report versions per domain with reasons for exclusion.

|  | **Self** | **Proxy** | *Reason for exclusion* |
| --- | --- | --- | --- |
| *Physical functions* |  | During the past month the individual was bothered by **dizziness** | - Frequency response options 'Don't know' & 'Not applicable' >30% |
|  | During the past month I was bothered by **problems eating** ***(f.e. eating too much or too little, eating unusual things)*** |  | - Local independence; This item is associated with the item 'problems with stools (f.e. constipation or diarrhea)'. In clinic, there is generally less focus on stool problems although constipation is a clinically relevant problem, according to the experts. |
|  | During the past month I was bothered by **problems with my hearing or ears** ***(f.e. infections, hearing loss)*** | During the past month the individual was bothered by **problems with hearing or ears *(f.e. infections, hearing loss)*** | - Frequency response option 'Not at all' >85% - Low factor loading |
|  | During the past month I was bothered by **heart or vascular problems *(f.e. rhythm abnormalities)*** | During the past month the individual was bothered by **heart or vascular problems *(f.e. rhythm abnormalities)*** | - Frequency response option 'Not at all' >85% |
|  | During the past month I was bothered by **spasticity *(high muscle tone)*** |  | - Frequency response option 'Not at all' >85% |
| *Mental functions* |  | During the past month the individual experienced **problems with certain skills *(f.e. arithmetic, reading, writing)*** | - Frequency response options 'Don't know' & 'Not applicable' >30% |
|  |  | During the past month the individual experienced **shyness** | - Frequency response options 'Don't know' & 'Not applicable' >30% |
|  |  | During the past month the individual experienced **difficulty with self-acceptance** | - Frequency response options 'Don't know' & 'Not applicable' >30% |
|  | During the past month I experienced **difficulty dealing with addictive substances *(f.e. alcohol, drugs, gaming)*** | During the past month the individual experienced **difficulty dealing with addictive substances *(f.e. alcohol, drugs, gaming)*** | - Low factor loading - Monotonicity - Additionally for proxy: Frequency response options 'Don't know' & 'Not applicable' >30% |
|  |  | During the past month the individual felt **unhappy, sad or depressed** | - Frequency response options 'Don't know' & 'Not applicable' >30% - These items rely on internal perception; difficult to estimate as a proxy |
|  |  | During the past month the individual felt **nervous or stressed** | - Frequency response options 'Don't know' & 'Not applicable' >30% - These items rely on internal perception; difficult to estimate as a proxy |
|  |  | During the past month the individual felt **anxious or scared** | - Frequency response options 'Don't know' & 'Not applicable' >30% - These items rely on internal perception; difficult to estimate as a proxy |
|  |  | During the past month the individual felt **lonely** | - Frequency response options 'Don't know' & 'Not applicable' >30% - These items rely on internal perception; difficult to estimate as a proxy |
|  | During the past month I **thought about killing myself (suicide)** | During the past month the individual **talked about killing him/herself (suicide)** | - Frequency response option 'Not at all' >85% |
|  |  | During the past month the individual **worried a lot** | - Frequency response options 'Don't know' & 'Not applicable' >30% |
|  | During the past month I **saw or heard things that other people did not see or hear *(f.e. hallucinations)*** |  | - Frequency response option 'Not at all' >85% |
|  | During the past month I **was physically aggressive towards others *(f.e. throwing things, kicking, hitting)*** |  | - Frequency response option 'Not at all' >85% |
|  | During the past month I **tried to hurt myself** |  | - Frequency response option 'Not at all' >85% |
|  |  | During the past month the individual worried about **tumor growth** | - Frequency response options 'Don't know' & 'Not applicable' >30% |
|  | During the past month I worried about **family planning *(f.e. passing on TSC)*** | During the past month the individual worried about **family planning *(f.e. passing on TSC)*** | - Low factor loading - Monotonicity - Additionally for proxy: Frequency response options 'Don't know' & 'Not applicable' >30% |
|  |  | During the past month the individual worried about **money *(f.e. due to being unable to work or absences during hospital visits)*** | - Frequency response options 'Don't know' & 'Not applicable' >30% |
|  |  | During the past month the individual worried about **financial independency** | - Frequency response options 'Don't know' & 'Not applicable' >30% |
|  |  | During the past month the individual worried about **social security *(f.e. reimbursement of devices or care)*** | - Frequency response options 'Don't know' & 'Not applicable' >30% |
| *Functioning in daily life* |  | During the past month the individual was limited in **planning and organizing** | - Frequency response options 'Don't know' & 'Not applicable' >30% |
|  | During the past month I was limited in **getting along with strangers** |  | - Local independence (similar to C8) |
|  | During the past month I was limited in **washing and dressing myself** |  | - Frequency response option 'Not at all' >85% |
|  | During the past month I was limited in **walking independently** |  | - Frequency response option 'Not at all' >85% |
|  | During the past month I was limited in **caring for my health *(f.e. taking medication)*** |  | - Frequency response option 'Not at all' >85% |
|  |  | During the past month the individual was limited in **his/her financial independency** | - Frequency response options 'Don't know' & 'Not applicable' >30% |
|  |  | During the past month the individual was limited in **making use of transportation *(f.e. driving a car, riding a bike, taking public transportation)*** | - Frequency response options 'Don't know' & 'Not applicable' >30% |
