# Supplementary material for: Understanding the impact of tuberous sclerosis complex: development and validation of the TSC-PROM
Source: BMC Med. 2023 Aug 8;21:298. doi: 10.1186/s12916-023-03012-4 (PMC10408092; doi:10.1186/s12916-023-03012-4)
Supplement: Supplementary file 2 — Additional file 2. TSC-PROM self and proxy version (English). The English self-report and proxy-report versions of the TSC-PROM. [file 12916_2023_3012_MOESM2_ESM.pdf]

TSC-PROM
Questionnaire for adults with Tuberous Sclerosis Complex (TSC)

Explanation and instructions

What is this questionnaire about?
You are filling in this questionnaire because you have Tuberous Sclerosis Complex.
This questionnaire is about the complaints and limitations you experience.

The questionnaire consists of the following domains:
- Baseline information
- Physical functions
- Mental functions
- Activities and participation
- Social support
- Quality of life

How to fill in this questionnaire?
Read the instructions with every section carefully.
Choose the answer that is most appropriate. Don't worry if some questions appear not to apply to you. We have to ask the same questions to everybody.

Explanation and examples
Some questions contain a short explanation. This explains the meaning of the mentioned term.
If f.e. (for example) is used, one or more examples follow. Possibly these examples are not applicable to you, but they may help you understand the question better.

Time
Filling in the questionnaire will take approximately 20 min.

 ©Van Eeghen

Baseline information

1. **What is your sex?**
   - ◻ Male
   - ◻ Female
   - ◻ Other

2. **What is your age?**
   _____years

3. **What is your nationality?**
   - ◻ American
   - ◻ Other (please specify): ______________

4. **At what age were you diagnosed with TSC?**
   _____years

5. **(a) Has genetic testing been performed?**
   - ◻ I don’t know (go to question 6)
   - ◻ No (go to question 6)
   - ◻ Yes

   (b) What were the results?
   - ◻ I don’t know
   - ◻ TSC1 mutation
   - ◻ TSC2 mutation
   - ◻ No mutation identified
   - ◻ Mutations found but uncertain if they cause TSC

6. **Which organs show, or have shown, symptoms of TSC? For example: tubers, tumors, pigment changes**
   - ◻ None
   - ◻ Skin
   - ◻ Lungs
   - ◻ Heart
   - ◻ Other, namely _____
   - ◻ Brain
   - ◻ Kidneys
   - ◻ Eyes
   - ◻ Mouth

7. **Do you use medication?**
   - ◻ No
   - ◻ Yes (please list all the medication you use) ____
     ____
     ____

 ©Van Eeghen

8. (a) Do you have epilepsy, now or in the past?

- ☐ No (go to question 9)
- ☐ Yes

(b) At what age did you have your first seizure?

_ _ _ _years and _ _ _ _months

- ☐ I don't know

(c) How often do you have seizures?

- ☐ Daily, approximately_ _ _ per day
- ☐ Weekly, approximately _ _ _ per week
- ☐ Monthly, approximately_ _ _per month
- ☐ I am seizure free, since _ _ _years old

(d) Do you have a vagal nerve stimulator

- ☐ No
- ☐ Yes

(e) Are you on a ketogenic diet?

- ☐ No
- ☐ Yes

9. What is your level of intellectual functioning

- ☐ Normal intellectual ability
- ☐ Normal intellectual ability with specific learning disability (dyscalculia, dyslexia)
- ☐ Mild or moderate intellectual disability
- ☐ I don't know

10. What was your last measured IQ or developmental age (if known)?

- ☐ My IQ was _ _ _ measured on_ _ _ _ _ _ _ _ _ _ (date or year)
- ☐ My developmental age was (approximately)_ _ _ _ _ measured on _ _ _ _ _ _ _ _ _ _ (date or year)
- ☐ I don't know

11. What is the highest level of education that you have completed?

- ☐ None
- ☐ Preschool/kindergarten
- ☐ Primary education
- ☐ Primary education, special education program
- ☐ Lower secondary education (Middle school or Junior High)
- ☐ Secondary education, special education program
- ☐ Upper to post-secondary (Senior High school, 1-year certificate programs)
- ☐ Academic higher education or doctoral (Bachelor, Master, PhD)

 ©Van Eeghen

12. What is your current living situation?

- ◻ I live alone, without assistance
- ◻ I live with other people, without assistance
- ◻ I live alone with ambulatory professional support
- ◻ I live with other people and with ambulatory professional support
- ◻ I live in an assisted living facility for people with a disability (no 24 hour care)
- ◻ I live in an assisted living facility for people with a disability (with 24 hour care)

13. Have you ever been diagnosed with any of the following?

	**No**	**Yes**	**I don't know**
Autism spectrum disorder (Autism, ASS, PDD-NOS, Asperger)	◻	◻	◻
Attention deficit hyperactivity disorder (ADD, ADHD)	◻	◻	◻
Obsessive compulsive disorder (OCD)	◻	◻	◻
Anxiety disorder	◻	◻	◻
Depressive disorder	◻	◻	◻
Psychotic disorder (f.e. schizophrenia)	◻	◻	◻

Other diagnoses, namely ______________________________________

14. (a) Do you have any other health concerns besides your TSC?

- ◻ No (go to question 15)
- ◻ Yes

(b) What are these health concerns?

- ◻ High blood pressure (hypertension)
- ◻ Diabetes ('sugar')
- ◻ Thyroid problems
- ◻ Malignant tumor (cancer)
- ◻ Other (please specify): ______________________________________

15. (a) In the past year, have you experienced any major life events?

- ◻ No (go to the next section)
- ◻ Yes

(b) What kind of life events?

- ◻ Moving house
- ◻ Change of employment / daytime occupation
- ◻ Severe illness or death of a family member or friend
- ◻ Another major life event, namely ______________________________

 ©Van Eeghen

Physical functions

In general, how would you rate your overall health? Please put a mark on the ruler below.

Very poor 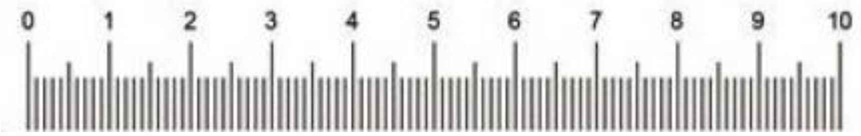 Excellent

Below are complaints or problems related to a person's physical functions that people with or without TSC may experience. Please indicate how much these complaints have troubled you during the last month. If any of the problems are always present, please include them in your estimation of your physical health in the past month.

Reply to each statement by ticking one box per row.

During the past month I was bothered by	A lot	Somewhat	A little	Not at all
1. difficulty sleeping				
2. fatigue				
3. dizziness				
4. problems with my weight *(f.e. unexpected weight loss or weight gain)*				
5. problems with my stomach *(f.e. acid reflux, vomiting, nausea)*				
6. problem with stools *(f.e. constipation or diarrhea)*				

During the past month I was bothered by	A lot	Somewhat	A little	Not at all
7. problems with my vision or eyes *(f.e. difficulty seeing, squinting)*				
8. speech and/or language problems (*f.e. stuttering, others having difficulty understanding my speech, unintelligible speech*)				
9. problems with my balance *(f.e. difficulty with stability when sitting, standing, or walking)*				
10. problems with my motor skills *(f.e. clumsiness, bad coordination)*				

©Van Eeghen

During the past month I was bothered by	A lot	Somewhat	A little	Not at all
11. skin abnormalities				
12. inflammation *(f.e. flu, respiratory infection, bladder infection, oral ulcers)*				
13. epileptic insults *(f.e. seizures, staring spells)*				
14. pain				
15. breathing problems *(f.e. shortness of breath, wheezing, coughing)*				
16. problems with my kidneys				
17. fluid retention (*f.e. ankle edema)*				
18. physical problems without a clear cause				

19. During last month I was bothered by side effects from my medication

□ No

□ Yes (please specify): --

 ©Van Eeghen

Mental functions

In general how was your mental health, including your mood and thinking facilities, during the last month? Please put a mark on the ruler below.

Very poor 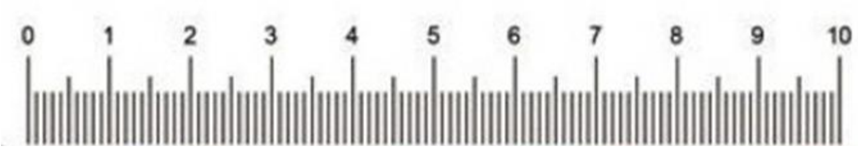 Excellent

Below are complaints or problems related to a person's mental functions that people with or without TSC may experience. Please indicate how much these complaints have troubled you during the last month. If any of the problems are always present, please include them in your estimation of your mental health in the past month.

Reply to each statement by ticking one box per row.

During the past month I experienced	A lot	Somewhat	A little	Not at all
1. overactive or hyperactive behavior				
2. restlessness *(f.e. fidgeting or squirming)*				
3. impulsivity *(f.e. doing or saying things without thinking)*				
4. difficulty concentrating or keeping my attention *(f.e. when reading or watching a movie)*				
5. difficulty remembering things				
6. difficulty with orientation in time or place *(f.e. knowing the date, knowing where I am)*				
7. problems with certain skills *(f.e. arithmetic, reading, writing)*				

During the past month I experienced	A lot	Somewhat	A little	Not at all
8. insecurity				
9. shyness				
10. difficulty making eye contact				
11. difficulty relating to peers				
12. difficulty identifying what someone was thinking or feeling				
13. difficulty estimating my own abilities and limitations				
14. difficulty to stand up for myself *(f.e. saying 'no')*				
15. difficulty to accept myself as I am				

 ©Van Eeghen

During the past month I experienced	A lot	Somew hat	A little	Not at all
16. difficulty in meeting new people				
17. difficulty with changes in routines				
18. hypersensitivity to sensory stimuli *(f.e. being touched, bright light, busy surroundings)*				
19. the need to repeatedly perform the same actions				
20. stubbornness				

During the past month I felt	A lot	Some what	A little	Not at all
21. unhappy, sad or depressed				
22. nervous or stressed				
23. anxious or scared				
24. lonely				

During the past month I	A lot	Some what	A little	Not at all
25. had mood swings				
26. had trouble handling stress				
27. panicked easily				
28. worried a lot				
29. couldn't get specific thoughts out of my head				
30. had temper tantrums				
31. was verbally aggressive towards others *(f.e. cursing, scolding)*				

During the past month I worried about	A lot	Some what	A little	Not at all
32. tumor growth				
33. epilepsy				
34. side effects of medication				
35. money *(f.e. due to being unable to work or absences during hospital visits)*				
36. my financial independence				
37. my social security *(f.e. reimbursement of devices or care)*				

 ©Van Eeghen

Activities and participation

During the past month, were you able to do your daily activities (with help as needed)?
Please put a mark on the ruler below.

Not at all 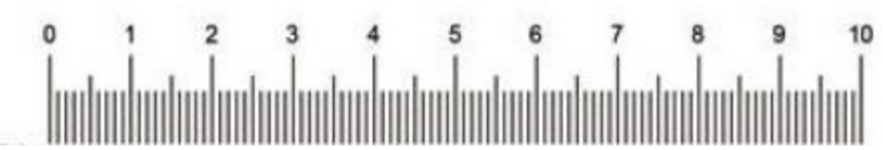 **Completely**

Below, activities are listed that occur in daily life for people with and without TSC. Please score how much you were hindered in performing these activities over the last month.

Reply to each statement by ticking one box per row.

During the past month I was limited in	Always	Often	Some-times	Never
1. communicating with others				
2. learning something new				
3. planning and organizing				
4. remembering things				
5. doing two things simultaneously *(multi-tasking)*				
6. getting along with people that I know well				
7. building a relationship/making friends				

During the past month I was limited in	Always	Often	Some-times	Never
8. participating in sport/physical exercise				
9. my financial independency				
10. making my own choices *(autonomy)*				
11. managing/planning my own free time				
12. participating in daily activities, work or internship				
13. making use of transportation *(f.e. driving a car, riding a bike, taking public transportation)*				

 ©Van Eeghen

Social support

In the past month, did you receive the kind of support that you needed?
Please put a mark on the ruler below.

Not at all 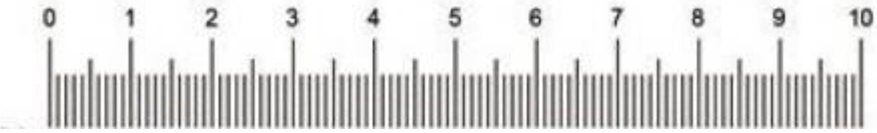

Completely

The following statements address how satisfied or dissatisfied you were with different aspects of your life in the past month.

Reply to each statement by ticking one box per row.

In the past month, I was satisfied with the support I received from	No, not at all	A little	Mostly	Completely	Not applicable
1. my family/partner					
2. my friends					
3. patient support groups (patient organisation)					
4. mental healthcare professionals *(f.e. psychiatrist, psychologist, social worker)*					
5. medical professionals *(f.e. doctors, nurses)*					
6. non-medical professionals *(f.e. caretakers)*					
7. from daycare activities, work or internship *(f.e. employer or colleague)*					

In the past month, I was satisfied with	No, not at all	A little	Mostly	Completely	Not applicable
8. how my medication is working					
9. the home where I live					
10. the availability of information about TSC					
11. my social relationships					
12. my sex life					
13. my finances					

 ©Van Eeghen

Quality of life

The last question asks about quality of life

How would you rate your quality of life over **the past month?**
Please put a mark on the ruler below.

Very poor 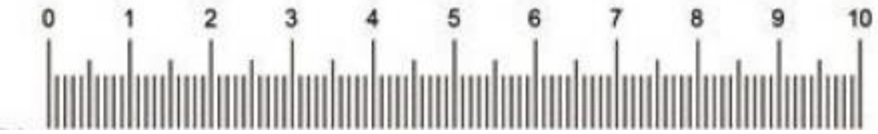

Excellent

This is the end of the questionnaire.

 ©Van Eeghen

TSC-PROM
Questionnaire for adults with Tuberous Sclerosis Complex (TSC)

Explanation and instructions

What is this questionnaire about?
You are filling in this questionnaire on behalf of your relative or client with Tuberous Sclerosis Complex.
This questionnaire is about the complaints and limitations he/she experiences.

The questionnaire consists of the following domains:
- Baseline information
- Physical functions
- Mental functions
- Activities and participation
- Social support
- Quality of life

How to fill in this questionnaire?
Read the instructions with every section carefully.
If possible, try and give the answer that you think your relative/client would give.
A statement may be for example: during the last month there was '*worrying or brooding'.* What matters is not if you were worried, but if your relative/client was worried.

Don't worry if some questions appear not to apply to your relative/client. We have to ask the same questions for everybody.
In the rest of the questionnaire we will call your relative/client 'the individual'.

Explanation and examples
Some questions contain a short explanation. This explains the meaning of the mentioned term.
If f.e. (for example) is used, one or more examples follow. Possibly these examples are not applicable to you, but they may help you understand the question better.

Time
Filling in the questionnaire will take approximately 20 min.

 ©Van Eeghen

Baseline information

1. What is the individual's sex?

- ◻ Male
- ◻ Female
- ◻ Other

2. What is the individual's age?

_____years

3. What is the individual's nationality?

- ◻ American
- ◻ Other (please specify): ______________

4. What is your relationship to the individual?

- ◻ Father
- ◻ Mother
- ◻ Brother
- ◻ Sister
- ◻ Caretaker
- ◻ Other (please specify): ______________

5. At what age was TSC diagnosed?

_____years

6. (a) Has genetic testing been performed?

- ◻ I don't know (go to question 6)
- ◻ No (go to question 6)
- ◻ Yes

(b) What were the results?

- ◻ I don't know
- ◻ TSC1 mutation
- ◻ TSC2 mutation
- ◻ No mutation identified
- ◻ Mutations found but uncertain if they cause TSC

7. Which organs show, or have shown, symptoms of TSC? For example: tubers, tumors, pigment changes

- ◻ None
- ◻ Skin
- ◻ Lungs
- ◻ Heart
- ◻ Other, namely _________
- ◻ Brain
- ◻ Kidneys
- ◻ Eyes
- ◻ Mouth

 ©Van Eeghen

8. Is the individual using medication?

- ◻ No
- ◻ Yes (please list all medication) ____
  ____
  ____

9. (a) Does the individual have epilepsy, now or in the past?

- ◻ No (go to question 9)
- ◻ Yes

(b) At what age did the first seizure occur?

____years and ____months

- ◻ I don't know

(c) How often do seizures occur?

- ◻ Daily, approximately___ per day
- ◻ Weekly, approximately___per week
- ◻ Monthly, approximately___per month
- ◻ The individual is seizure free, since___ years old

(d) Does the individual have a vagal nerve stimulator

- ◻ No
- ◻ Yes

(e) Is the individual on a ketogenic diet?

- ◻ No
- ◻ Yes

10. What is the (estimated) level of intellectual functioning of the individual?

- ◻ Normal intellectual ability
- ◻ Normal intellectual ability with specific learning disability (dyscalculia, dyslexia)
- ◻ Mild or moderate intellectual disability
- ◻ Severe or profound intellectual disability
- ◻ I don't know

11. What was the last measured IQ or developmental age of the individual?

- ◻ The IQ was ___ measured on _______ (date or year)
- ◻ The developmental age was (approximately)_____ measured on _______ (date or year)
- ◻ I don't know

12. What is the highest level of education the individual completed?

- ◻ None
- ◻ Preschool/kindergarten
- ◻ Primary education
- ◻ Primary education, special education program

 ©Van Eeghen

- ◻ Lower secondary education (Middle school or Junior High)
- ◻ Secondary education, special education program
- ◻ Upper to post-secondary (Senior High school, 1-year certificate programs)
- ◻ Academic higher education or doctoral (Bachelor, Master, PhD)
- ◻ I don't know

13. What is the current living situation of the individual?

- ◻ Alone, without assistance
- ◻ With other people, without assistance
- ◻ Alone with ambulatory professional support
- ◻ With other people and with ambulatory professional support
- ◻ In an assisted living facility for people with a disability (no 24 hour care)
- ◻ In an assisted living facility for people with a disability (with 24 hour care)

14. Has the individual ever been diagnosed with any of the following?

	**No**	**Yes**	**I don't know**
Autism spectrum disorder (Autism, ASS, PDD-NOS, Asperger)	◻	◻	◻
Attention deficit hyperactivity disorder (ADD, ADHD)	◻	◻	◻
Obsessive compulsive disorder (OCD)	◻	◻	◻
Anxiety disorder	◻	◻	◻
Depressive disorder	◻	◻	◻
Psychotic disorder (f.e. schizophrenia)	◻	◻	◻

Other diagnoses, namely ______________________________________

15. (a) Does the individual has any other health concerns besides TSC?

- ◻ No (go to question 15)
- ◻ Yes

(b) What are these health concerns?

- ◻ High blood pressure (hypertension)
- ◻ Diabetes ('sugar')
- ◻ Thyroid problems
- ◻ Malignant tumor (cancer)
- ◻ Other (please specify):______________________________________

16. (a) In the past year, has the individual experienced any major life events?

- ◻ No (go to the next section)
- ◻ Yes

(b) What kind of life events?

- ◻ Moving house
- ◻ Change of employment / daytime occupation
- ◻ Severe illness or death of a relative or friend
- ◻ Another major life event, namely ______________________________

 ©Van Eeghen

Physical functions

In general, how was the physical health of the individual over the past month? Please put a mark on the ruler below.

Very poor 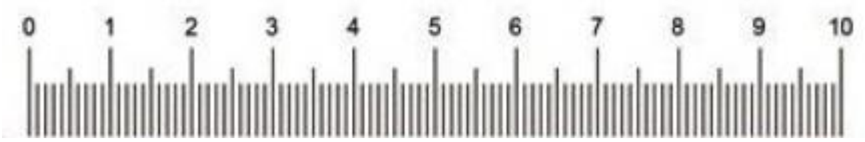 **Excellent**

Below are complaints or problems related to a person’s physical functions that people with or without TSC may experience. Please indicate how much these complaints have troubled the individual during the last month. If any of the problems are always present, please include them in your estimation of the physical health in the past month.

Reply to each statement by ticking one box per row.

During the past month the individual was bothered by	A lot	Somehwat	A little	Not at all	I don’ t know
1. difficulty sleeping					
2. fatigue					
3. problems eating *(f.e.eating too much or too little, eating unusual things)*					
4. problems with his/her weight *(f.e. unexpected weight loss or weight gain)*					
5. problems with his/her stomach *(f.e. acid reflux, vomiting, nausea)*					
6. problem with his/her stool *(f.e. constipation or diarrhea)*					

During the past month the individual was bothered by	A lot	Somehwat	A little	Not at all	I don’ t know
7. problems with vision or eyes *(f.e. difficulty seeing, squinting)*					
8. speech and/or language problems (*f.e. stuttering, others having difficulty understanding his/her speech, unintelligible speech*)					
9. problems with the equilibrium *(f.e. balance problems, difficulty with stability when sitting, standing, walking)*					
10. problems with motor skills *(f.e. clumsiness, bad coordination)*					

©Van Eeghen

During the past month the individual was bothered by	A lot	Somehwat	A little	Not at all	I don’ t know
11. skin abnormalities					
12. inflammation *(f.e. flu, respiratory infection, bladder infection, mouth ulcers)*					
13. epileptic insults *(f.e. seizures, staring spells)*					
14. pain					
15. breathing problems *(f.e. shortness of breath, wheezing, coughing)*					
16. problems with the kidneys					
17. spasticity (*high muscle tone*)					
18. fluid retention (*f.e. ankle edema)*					
19. physical problems without a clear cause					

20. <u>During last month the individual was bothered by side effects from the medication</u>
   - ◻ No
   - ◻ Yes (please specify):---

 ©Van Eeghen

Mental functions

In general how was the mental health of the individual, including your mood and thinking facilities, during the last month?
Please put a mark on the ruler below.

Very poor 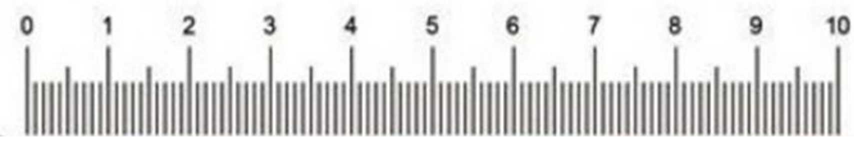

Excellent

Below are complaints or problems related to a person’s mental functions that people with or without TSC may experience. Please indicate how much these complaints have troubled the individual during the last month. If any of the problems are always present, please include them in your estimation of the mental health in the past month.

Reply to each statement by ticking one box per row.

During the past month the individual experienced	A lot	Somewhat	A little	Not at all	I don’ t know	Not applicable
1. overactive or hyperactive behavior						
2. restlessness *(f.e. fidgeting or squirming)*						
3. impulsivity *(f.e. doing or saying things without thinking)*						
4. difficulty concentrating or keeping attention *(f.e. when reading or watching a movie)*						
5. difficulty remembering things						
6. difficulty with orientation in time or place *(f.e. knowing the date, knowing where he/she is)*						

During the past month the individual experienced	A lot	Somewhat	A little	Not at all	I don’ t know	Not applicable
7. insecurity						
8. difficulty making eye contact						
9. difficulty relating to peers						
10. difficulty identifying what someone was thinking or feeling						
11. difficulty estimating his/her own abilities and limitations						
12. difficulty to stand up for him/herself *(f.e. saying ‘no’)*						

©Van Eeghen

<u>During the past month the individual experienced</u>	A lot	Somewhat	A little	Not at all	I don't know	Not applicable
13. difficulty meeting new people						
14. difficulty with changes in routines						
15. hypersensitivity to sensory stimuli *(f.e. being touched, bright light, busy surroundings)*						
16. the need to repeatedly perform the same actions						
17. stubbornness						

<u>During the past month the individual</u>	A lot	Somewhat	A little	Not at all	I don't know	Not applicable
18. had moodswings						
19. had trouble handling stress						
20. panicked easily						
21. saw or heard things that other people did not see or hear *(f.e. hallucinations)*						
22. couldn't get specific thoughts out of his/her head						
23. had temper tantrums						
24. was physically aggressive towards others *(f.e. throwing things, kicking, hitting)*						
25. was verbally aggressive towards others *(f.e. cursing, scolding)*						
26. tried to hurt him/herself						

<u>During the past month the individual worried about</u>	A lot	Somewhat	A little	Not at all	I don't know	Not applicable
27. epilepsy						
28. side effects of medication						

©Van Eeghen

Activities and participation

During the past month, was the individual able to do his/her daily activities (with help as needed)?
Please put a mark on the ruler below.

Not at all 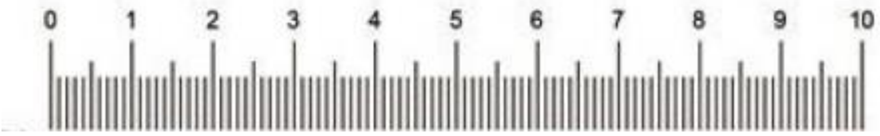

Completely

Below, activities are listed that occur in daily life for people with and without TSC. Please score how much the individual was hindered in performing these activities over the last month.

Reply to each statement by ticking one box per row.

During the past month the individual was limited in	Always	Often	Sometimes	Never	I don’ t know	Not applicable
1. communicating with others						
2. learning something new						
3. remembering things						
4. doing two things simultaneously *(multi-tasking)*						
5. getting along with people that he/she knows well						
6. getting along with strangers						
7. building a relationship/making friends						

During the past month the individual was limited in	Always	Often	Sometimes	Never	I don’ t know	Not applicable
8. washing and dressing him/herself						
9. walking independently						
10. participating in sport/physical exercise						
11. caring for his/her health *(f.e. taking medication)*						
12. making his/her own choices *(autonomy)*						
13. managing/planning his/her own free time						
14. participating in daily activities, work or internship						

©Van Eeghen

Social support

In the experience of the individual, did he/she receive the needed support during the last month?
Please put a mark on the ruler below.

Not at all 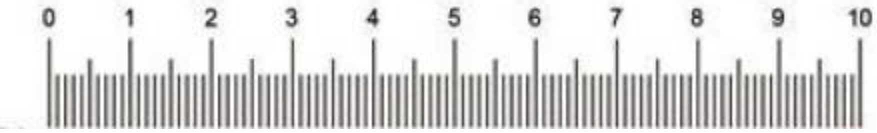

Completely

The following statements address how satisfied or dissatisfied the individual seems to be with different aspects of his/her life in the past month.

Reply to each statement by ticking one box per row.

In the past month, the individual seems to be satisfied with the support he/she received from	No, not at all	A little	Mostly	Completely	I don’ t know	Not applicable
1. family/partner						
2. friends						
3. patient support groups (patient organisation)						
4. mental healthcare professionals *(f.e. psychiatrist, psychologist, social worker)*						
5. medical professionals *(f.e. doctors, nurses)*						
6. non-medical professionals *(f.e. caretakers)*						
7. from daycare activities, work or internship *(f.e. employer or colleague)*						

In the past month, the individual seems to be satisfied with	No, not at all	A little	Mostly	Completely	I don’ t know	Not applicable
8. how the medication is working						
9. the home where he/she lives						
10. the availability of information about TSC						
11. his/her social relationships						
12. his/her sex life						
13. his/her finances						

©Van Eeghen

Quality of life

The last question asks about quality of life

How do you think the individual would rate his/her quality of life over **the past month?**
Please put a mark on the ruler below.

Very poor 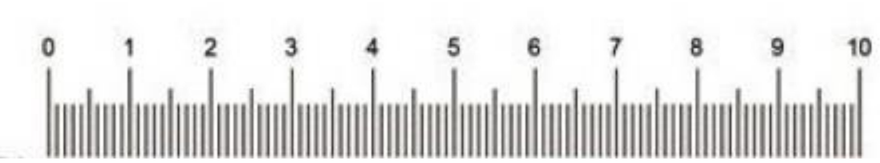 **Excellent**

This is the end of the questionnaire.

 ©Van Eeghen
