# Supplementary material for: Understanding the impact of tuberous sclerosis complex: development and validation of the TSC-PROM
Source: BMC Med. 2023 Aug 8;21:298. doi: 10.1186/s12916-023-03012-4 (PMC10408092; doi:10.1186/s12916-023-03012-4)
Supplement: Supplementary file 3 — Additional file 3. TSC-PROM self and proxy version (Dutch). The Dutch self-report and proxy-report versions of the TSC-PROM. [file 12916_2023_3012_MOESM3_ESM.pdf]

TSC-PROM
Vragenlijst voor volwassenen met Tubereuze Sclerose Complex (TSC)

Uitleg en instructies

Waar gaat de vragenlijst over?
U vult deze vragenlijst in omdat u Tubereuze Sclerose Complex heeft.
De vragenlijst gaat over de klachten en beperkingen die u ervaart.

De vragenlijst bestaat uit de volgende delen:

- Basisinformatie
- Lichamelijk functioneren
- Geestelijk functioneren
- Activiteiten en participatie
- Sociale steun
- Kwaliteit van leven

Hoe moet u de vragenlijst invullen?
Lees de instructies bij elk onderdeel zorgvuldig.
Kies het antwoord dat het meest bij u past.
Maakt u zich geen zorgen als sommige vragen niet op u van toepassing zijn. We stellen iedereen dezelfde vragen.

Toelichting en voorbeelden
Bij sommige vragen staat een korte toelichting. Hierin leggen we uit wat we bedoelen met de genoemde term.
Als er 'bijv.' staat, volgen er één of meerdere voorbeelden. Mogelijk zijn deze voorbeelden niet op u van toepassing, maar helpen ze u de vraag beter te begrijpen.

Tijdsduur
Het invullen van de vragenlijst zal ongeveer 20 minuten duren.

©Van Eeghen

Basisinformatie

1. Wat is uw geslacht?

- ☐ Man
- ☐ Vrouw
- ☐ Anders

2. Wat is uw leeftijd?

_ _ _ _ _ Jaar _ _ _ _ _ maanden

3. Wat is uw nationaliteit?

- ☐ Nederlands
- ☐ Belgisch
- ☐ Anders, namelijk _ _ _ _ _ _ _ _ _ _ _ _ _ _

4. Op welke leeftijd werd de diagnose TSC gesteld?

_ _ _ _ _ jaar

5. (a) Is er genetisch onderzoek gedaan?

- ☐ Weet niet (ga naar vraag 6)
- ☐ Nee (ga naar vraag 6)
- ☐ Ja

(b) Wat was hiervan de uitslag?

- ☐ Weet niet
- ☐ TSC1 mutatie
- ☐ TSC2 mutatie
- ☐ Geen afwijkingen gevonden
- ☐ Wel een mutatie gevonden, maar niet zeker of dit de ziekte veroorzaakt

6. In welke organen zijn symptomen van TSC aanwezig, of aanwezig geweest?
Bijv. tubers, tumoren, pigmentafwijkingen

- ☐ Geen
- ☐ Huid
- ☐ Longen
- ☐ Hart
- ☐ Hersenen
- ☐ Nieren
- ☐ Ogen
- ☐ Mond
- ☐ Overig, namelijk _ _ _ _ _ _ _ _ _

7. Gebruikt u medicatie?

- ☐ Nee
- ☐ Ja, namelijk (graag alle huidige medicatie noteren) _

_ _

_ _

©Van Eeghen

8. (a) Heeft u epilepsie (of in het verleden gehad)?

- ◻ Nee (ga naar vraag 9)
- ◻ Ja

(b) Op welke leeftijd had u uw eerste aanval?

_____ jaar en _____ maanden

(c) Hoe vaak komen insulten voor?

- ◻ Dagelijks, ongeveer _____ per dag
- ◻ Wekelijks, ongeveer _____ per week
- ◻ Maandelijks, ongeveer _____ per maand
- ◻ Jaarlijks, ongeveer _____ per jaar
- ◻ Ik ben aanvalsvrij, sinds de leeftijd van_____ jaar

(d) Heeft u een nervus vagus stimulator?

- ◻ Nee
- ◻ Ja

(e) Volgt u een ketogeen dieet?

- ◻ Nee
- ◻ Ja

9. Wat is uw niveau van functioneren?

- ◻ Normaal begaafd of bovengemiddeld begaafd
- ◻ Normaal begaafd met specifieke leerproblemen (zoals dyslexie of dyscalculie)
- ◻ Lichte of matige verstandelijke beperking

10. Wat is uw laatst gemeten IQ of ontwikkelingsleeftijd (indien bekend)?

- ◻ Het IQ was _____ gemeten op___________ (datum of jaartal)
- ◻ De ontwikkelingsleeftijd was (ongeveer) _____ gemeten op ___________ (datum of jaartal)
- ◻ Weet niet

11. Wat is uw hoogste afgeronde opleiding?

<u>Nederland</u>

- ◻ Geen
- ◻ Basisschool/ Lagere school regulier onderwijs
- ◻ Basisschool/ Lagere school speciaal onderwijs
- ◻ Lager beroepsonderwijs (bijv. huishoudschool, LTS, LEAO, LHNO, praktijkonderwijs)
- ◻ Speciaal voortgezet onderwijs
- ◻ Middelbaar algemeen voortgezet onderwijs (bijv. MAVO, IVO, (M)ULO, VMBO)
- ◻ Middelbare beroepsopleiding (bijv. MBO 2-3, MTS, MEAO, MHNO, INAS)
- ◻ Hoger algemeen en voorbereidend wetenschappelijk onderwijs (bijv. HAVO, VWO, HBS, MMS, Gymnasium, Atheneum)

 ©Van Eeghen

- ◻ Hoger beroepsonderwijs (bijv. HBO, HTS, HEAO, HHMO)
- ◻ Wetenschappelijk onderwijs (bijv. Bachelor, Master, Master na Master, doctoraat)

België

- ◻ Geen
- ◻ Basisschool/ Lagere school regulier onderwijs
- ◻ Basisschool/ Lagere school buitengewoon onderwijs
- ◻ Lager secundair onderwijs (1ste graad SO)
- ◻ Buitengewoon secundair onderwijs
- ◻ Hoger secundair en post-secundair onderwijs (bijv. ASO, TSO, BSO)
- ◻ Professional bachelor/master
- ◻ Academic master of doctoraat

12. Waar woont u?

- ◻ Ik woon alleen, zonder begeleiding
- ◻ Ik woon met anderen, zonder begeleiding
- ◻ Ik woon alleen en krijg ambulante begeleiding
- ◻ Ik woon met anderen en krijg ambulante begeleiding
- ◻ Ik woon in een huis voor mensen met een beperking (géén 24-uurs zorg)
- ◻ Ik woon in een huis voor mensen met een beperking (24 uurs zorg)

13. Kreeg u, naast de diagnose TSC, ooit één of meerdere van onderstaande diagnoses?

	**Nee**	**Ja**	**Weet niet**
Autisme spectrum stoornis (Autisme, ASS, PDD-NOS, Asperger)	◻	◻	◻
Aandachtstekort-hyperactiviteitstoornis (ADD, ADHD)	◻	◻	◻
Obsessieve-compulsieve stoornis (OCD)	◻	◻	◻
Angststoornis	◻	◻	◻
Depressieve stoornis	◻	◻	◻
Psychotische stoornis (bijv. schizofrenie)	◻	◻	◻

Andere diagnose(s), namelijk ___________________________________

14. (a) Heeft u, naast de diagnose TSC, nog andere gezondheidsproblemen?

- ◻ Nee (ga naar vraag 15)
- ◻ Ja

(b) Welke gezondheidsproblemen heeft u?

- ◻ Hoge bloeddruk
- ◻ Suikerziekte (diabetes)
- ◻ Schildklieraandoening
- ◻ Kwaadaardige tumor (kanker)
- ◻ Anders, namelijk ___________________________________

 ©Van Eeghen

15. (a) Heeft u in het afgelopen jaar een ingrijpende gebeurtenis meegemaakt?

- ◻ Nee (ga naar het volgende hoofdstuk)
- ◻ Ja

(b) Wat voor ingrijpends heeft plaatsgevonden?

- ◻ Verhuizing
- ◻ Verandering van werk
- ◻ Ernstige ziekte of overlijden van familie/kennis
- ◻ Een andere gebeurtenis, namelijk ___________________________________

 ©Van Eeghen

Lichamelijk functioneren

Hoe vindt u over het algemeen uw lichamelijke gezondheid? Plaats een kruisje op de liniaal hieronder.

Zeer slecht 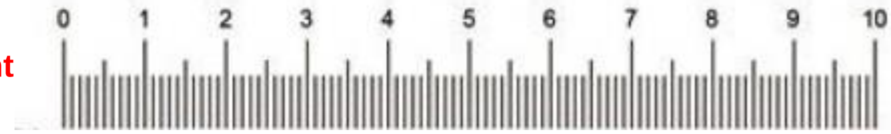 **Uitstekend**

Hieronder volgen klachten of problemen op het gebied van het lichamelijk functioneren die mensen (met of zonder TSC) kunnen ervaren. Wilt u scoren hoeveel last u heeft gehad van deze klachten gedurende de afgelopen maand? Wanneer de klachten altijd aanwezig zijn, zijn deze dus ook van toepassing op de afgelopen maand.

Geef een reactie op elke uitspraak door per rij één hokje aan te vinken.

Gedurende de afgelopen maand had ik last van:	Heel erg	Nogal	Een beetje	Helemaal niet
1. moeite met slapen				
2. moeheid				
3. duizeligheid				
4. problemen met het gewicht *(bijv. onbedoeld afvallen of aankomen)*				
5. maagklachten *(bijv. maagzuur, braken, misselijkheid)*				
6. problemen met de ontlasting *(bijv. verstopping of diarree)*				

Gedurende de afgelopen maand had ik last van:	Heel erg	Nogal	Een beetje	Helemaal niet
7. problemen met zien of de ogen *(bijv. niet goed kunnen zien, scheelzien)*				
8. spraak- en/of taalproblemen *(bijv. stotteren, moeilijk verstaanbaar zijn, moeite met woorden vinden)*				
9. problemen met het evenwicht *(bijv. balansproblemen, moeite met stabiel staan, lopen of zitten)*				
10. problemen van de motoriek *(bijv. onhandigheid, slechte coördinatie, stijfheid)*				

 ©Van Eeghen

Gedurende de afgelopen maand had ik last van:	Heel erg	Nogal	Een beetje	Helemaal niet
11. huidafwijkingen				
12. ontstekingen of infecties *(bijv. griep, luchtweginfectie, blaasontsteking, aften in de mond)*				
13. epileptische aanvallen *(bijv. trekkingen, staaraanvallen)*				
14. pijn				
15. ademhalingsproblemen *(bijv. kortademigheid, piepen, hoesten)*				
16. nierproblemen				
17. vochtophoping (*bijv. enkeloedeem*)				
18. lichamelijke problemen zonder duidelijke oorzaak				

19. Gedurende de afgelopen maand had ik last van bijwerkingen van de medicatie:

□ Nee

□ Ja, namelijk --

 ©Van Eeghen

<u>Geestelijk functioneren</u>

Hoe zou u over het algemeen uw geestelijke gezondheid, inclusief uw stemming en denkvermogen, beoordelen? Plaats een kruisje op de liniaal hieronder.

Zeer slecht 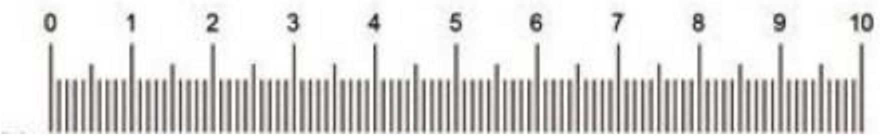

Uitstekend

Hieronder volgen klachten of problemen op het gebied van het geestelijk functioneren die mensen (met of zonder TSC) kunnen ervaren. Wilt u scoren hoeveel last u heeft gehad van deze klachten gedurende de afgelopen maand? Wanneer de klachten altijd aanwezig zijn, zijn deze dus ook van toepassing op de afgelopen maand.

Geef een reactie op elke uitspraak door per rij één hokje aan te vinken.

<u>Gedurende de afgelopen maand had ik:</u>	Heel erg	Nogal	Een beetje	Helemaal niet
1. last van overactief of hyperactief gedrag				
2. last van rusteloos of plukkerig gedrag (*bijv. wriemelen of wiebelen*)				
3. last van impulsief gedrag (*bijv. dingen doen of zeggen zonder na te denken*)				
4. moeite met concentreren of lang de aandacht bij iets te houden *(bijv. tijdens lezen of een film kijken)*				
5. moeite met het herinneren van dingen				
6. moeite met het oriënteren in tijd en plaats (*bijv. datum kennen, weten waar u bent)*				
7. moeite met bepaalde vaardigheden *(bijv. rekenen, lezen, schrijven)*				

<u>Gedurende de afgelopen maand had ik:</u>	Heel erg	Nogal	Een beetje	Helemaal niet
8. last van onzekerheid				
9. last van verlegenheid				
10. moeite met oogcontact maken				
11. moeite met leeftijdsgenoten om te gaan				
12. moeite met begrijpen wat iemand denkt of voelt				
13. moeite met mijn eigen mogelijkheden en beperkingen in te schatten				
14. moeite met voor mezelf opkomen *(bijv. nee zeggen*)				
15. moeite met mezelf te aanvaarden *(zelfacceptatie)*				

©Van Eeghen

Gedurende de afgelopen maand had ik:	Heel erg	Nogal	Een beetje	Helemaal niet
16. moeite met nieuwe mensen ontmoeten				
17. moeite met veranderen van routines				
18. last van overgevoeligheid voor prikkels *(bijv. aanrakingen, fel licht, een drukke omgeving)*				
19. last van het willen herhalen van eenzelfde handeling				
20. last van koppigheid of stuursheid				

Gedurende de afgelopen maand voelde ik me:	Heel erg	Nogal	Een beetje	Helemaal niet
21. ongelukkig, verdrietig of gedeprimeerd				
22. zenuwachtig of gespannen				
23. angstig of bang				
24. eenzaam				

Gedurende de afgelopen maand:	Heel erg	Nogal	Een beetje	Helemaal niet
25. kon mijn stemming of gevoel plotseling veranderen				
26. vond ik het moeilijk om te gaan met stress				
27. raakte ik snel in paniek				
28. piekerde ik veel				
29. kon ik bepaalde gedachten moeilijk uit mijn hoofd zetten				
30. had ik driftbuien				
31. was ik verbaal agressief naar anderen *(bijv. vloeken, schelden)*				

Gedurende de afgelopen maand maakte ik me zorgen over:	Heel erg	Nogal	Een beetje	Helemaal niet
32. tumorgroei				
33. epilepsie				
34. bijwerkingen van medicatie				
35. geld *(door bijv. niet kunnen werken of verzuim door ziekenhuisbezoeken)*				

 ©Van Eeghen

36. mijn financiële zelfstandigheid				
37. sociale zekerheid *(bijv. vergoeding van hulpmiddelen of zorg)*				

©Van Eeghen

Activiteiten en participatie

Kon u in de afgelopen maand uw dagelijkse activiteiten ondernemen (eventueel met hulp)?
Plaats een kruisje op de liniaal hieronder.

Helemaal niet 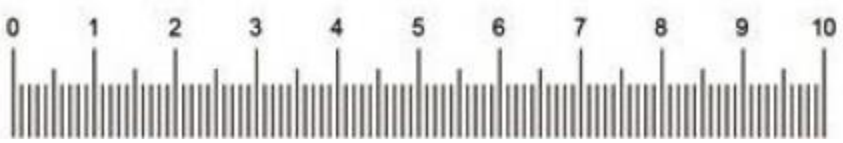

Volledig

Hieronder volgen activiteiten die plaatsvinden in het dagelijks leven van mensen met en zonder TSC. Wilt u scoren hoeveel belemmeringen u heeft ondervonden gedurende de afgelopen maand met het uitvoeren van deze activiteiten?

Geef een reactie op elke uitspraak door per rij één hokje aan te vinken.

Gedurende de afgelopen maand was ik belemmerd in:	Altijd	Vaak	Soms	Nooit
1. communicatie met anderen				
2. iets nieuws leren				
3. dingen plannen en organiseren				
4. dingen onthouden				
5. twee dingen tegelijkertijd doen (multi-tasking)				
6. omgaan met mensen die ik goed ken				
7. een band opbouwen met iemand of vrienden maken				

Gedurende de afgelopen maand was ik belemmerd in:	Altijd	Vaak	Soms	Nooit
8. sporten of aan lichaamsbeweging doen				
9. mijn financiële zelfstandigheid				
10. zelf te kunnen bepalen wat ik doe *(autonomie)*				
11. mijn eigen vrije tijd plannen/indelen				
12. deelname aan werk, stage of dagbesteding				
13. gebruikmaken van vervoermiddelen zoals auto, fiets of openbaar vervoer				

©Van Eeghen

<u>Sociale steun</u>

Kreeg u in de afgelopen maand het soort steun dat u nodig had?
Plaats een kruisje op de liniaal hieronder.

Helemaal niet 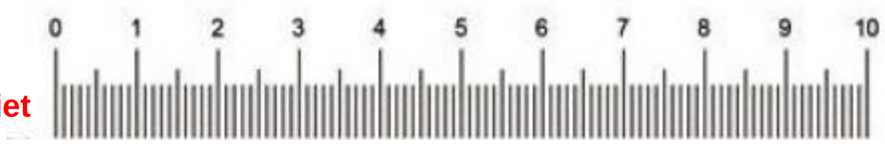

Volledig

De volgende uitspraken gaan over hoe tevreden u in de afgelopen maand was met verschillende aspecten van uw leven.

Geef een reactie op elke uitspraak door per rij één hokje aan te vinken.

<u>Ik was in de afgelopen maand tevreden over de steun die ik kreeg van:</u>	Helemaal niet	Enigszins	In grote mate	Volledig	Niet van toepassing
1. mijn familie/partner					
2. mijn vrienden					
3. de patiëntenvereniging					
4. hulpverleners uit de Geestelijke Gezondheidszorg *(bijv. psychiater, psycholoog, maatschappelijk werker)*					
5. medische hulpverleners (*bijv. artsen en verpleegkundigen*)					
6. niet-medische hulpverlening (*bijv. begeleiders)*					
7. werk, stage of dagbesteding *(bijv. collega of werkgever)*					

<u>Ik was in de afgelopen maand tevreden over:</u>	Helemaal niet	Enigszins	In grote mate	Volledig	Niet van toepassing
8. de werking van mijn medicijnen					
9. het huis waar ik woon					
10. de beschikbaarheid van informatie over TSC					
11. mijn sociale relaties					
12. mijn seksuele leven					
13. mijn financiële middelen					

 ©Van Eeghen

Kwaliteit van leven

De laatste vraag gaat over kwaliteit van leven.

Hoe zou u uw kwaliteit van leven beoordelen **in de afgelopen maand**?
Plaats een kruisje op de liniaal hieronder.

Zeer slecht

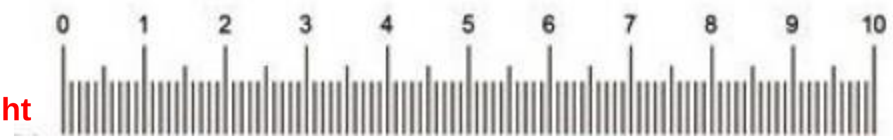

Uitstekend

Dit is het einde van de vragenlijst.

 ©Van Eeghen

TSC-PROM
Vragenlijst voor volwassenen met Tubereuze Sclerose Complex (TSC)

Uitleg en instructies

Waar gaat de vragenlijst over?
U vult deze vragenlijst in voor uw familielid of cliënt met Tubereuze Sclerose Complex.
De vragenlijst gaat over de klachten en beperkingen die hij/zij in het dagelijks leven ervaart.

De vragenlijst bestaat uit de volgende onderdelen:

- Basisinformatie
- Lichamelijk functioneren
- Geestelijk functioneren
- Activiteiten en participatie
- Sociale steun
- Kwaliteit van leven

Hoe moet u de vragenlijst invullen?
Lees de instructies bij elk onderdeel zorgvuldig.
Probeer het antwoord te geven waarvan u denkt dat uw familielid/cliënt zelf zou geven.
Een stelling kan bijvoorbeeld zijn: Gedurende de afgelopen maand was er sprake van *'zich zorgen maken of piekeren'*. Het gaat er dan niet om of u zich zorgen maakte, maar of uw familielid/cliënt zich zorgen maakte.
Maakt u zich geen zorgen als sommige vragen niet op uw familielid/cliënt van toepassing zijn. We moeten iedereen dezelfde vragen stellen.
In de rest van de vragenlijst noemen we 'uw familielid/cliënt' steeds 'betrokkene'.

Toelichting en voorbeelden
Bij sommige vragen staat een korte toelichting. Hierin leggen we uit wat we bedoelen met de genoemde term.
Als er 'Bijv.' staat, volgen er één of meerdere voorbeelden. Mogelijk zijn deze voorbeelden niet op betrokkene van toepassing, maar helpen ze u de vraag beter te begrijpen.

Tijdsduur
Het invullen van de vragenlijst zal ongeveer 20 minuten duren.

 ©Van Eeghen

Basisinformatie

1. Wat is het geslacht van betrokkene?

- ☐ Man
- ☐ Vrouw
- ☐ Anders

2. Wat is de leeftijd van betrokkene?

_ _ _ _ _ jaar

3. Wat is de nationaliteit van betrokkene?

- ☐ Nederlands
- ☐ Belgisch
- ☐ Anders, namelijk …

4. Wat is uw relatie tot de betrokkene?

- ☐ Vader
- ☐ Moeder
- ☐ Broer
- ☐ Zus
- ☐ (Persoonlijk) begeleider
- ☐ Anders, namelijk _

5. Op welke leeftijd werd de diagnose TSC gesteld?

_ _ _ _ _ Jaar

6. (a)Is er genetisch onderzoek gedaan bij betrokkene?

- ☐ Weet niet (ga naar vraag 7)
- ☐ Nee (ga naar vraag 7)
- ☐ Ja

(b) Wat was hiervan de uitslag?

- ☐ Weet niet
- ☐ TSC1 mutatie
- ☐ TSC2 mutatie
- ☐ Geen afwijkingen gevonden
- ☐ Wel een mutatie gevonden, maar niet zeker of dit de ziekte veroorzaakt

7. In welke organen zijn symptomen van TSC aanwezig, of aanwezig geweest? Bijv. tubers, tumoren, pigmentafwijkingen

- ☐ Geen
- ☐ Huid
- ☐ Longen
- ☐ Hart
- ☐ Hersenen
- ☐ Nieren
- ☐ Ogen
- ☐ Mond
- ☐ Overig, namelijk _ _ _ _ _ _ _ _ _ _ _ _

 ©Van Eeghen

8. Gebruikt betrokkene medicatie?

- ◻ Nee
- ◻ Ja, namelijk (graag alle huidige medicatie noteren) _
  _
  _

9. (a) Heeft betrokkene epilepsie (of in het verleden gehad)?

- ◻ Nee
- ◻ Ja

(b) Op welke leeftijd had betrokkene de eerste aanval?

_ _ _ _ _ jaar en _ _ _ _ _ maanden

- ◻ Weet niet

(c) Hoe vaak komen insulten voor?

- ◻ Dagelijks, ongeveer _ _ _ _ _ per dag
- ◻ Wekelijks, ongeveer _ _ _ _ _ per week
- ◻ Maandelijks, ongeveer _ _ _ _ _ per maand
- ◻ Jaarlijks, ongeveer _ _ _ _ _ per jaar
- ◻ Betrokkene is aanvalsvrij, sinds de leeftijd van _ _ _ _ _ jaar

(d) Heeft betrokkene een nervus vagus stimulator?

- ◻ Nee
- ◻ Ja

(e) Volgt betrokkene een ketogeen dieet?

- ◻ Nee
- ◻ Ja

10. Hoe zou u het functioneren van betrokkene inschatten?

- ◻ Normaal begaafd of bovengemiddeld begaafd
- ◻ Normaal begaafd met specifieke leerproblemen (zoals dyslexie of dyscalculie)
- ◻ Milde of matige verstandelijke beperking
- ◻ Ernstige of diepe verstandelijke beperking

11. Wat is het laatst gemeten IQ of de ontwikkelingsleeftijd (indien bekend) van betrokkene?

- ◻ Het IQ was _ _ _ _ _ gemeten op _ _ _ _ _ _ _ _ _ _ _ _ (datum of jaartal)
- ◻ De ontwikkelingsleeftijd was _ _ _ _ _ gemeten op _ _ _ _ _ _ _ _ _ _ _ _ (datum of jaartal)
- ◻ Weet niet

 ©Van Eeghen

12. Wat is de hoogste afgeronde opleiding van betrokkene?

Nederland

- ☐ Geen
- ☐ Basisschool/ Lagere school regulier onderwijs
- ☐ Basisschool/ Lagere school speciaal onderwijs
- ☐ Lager beroepsonderwijs (bijv. huishoudschool, LTS, LEAO, LHNO, praktijkonderwijs)
- ☐ Speciaal voortgezet onderwijs
- ☐ Middelbaar algemeen voortgezet onderwijs (bijv. MAVO, IVO, (M)ULO, VMBO)
- ☐ Middelbare beroepsopleiding (bijv. MBO 2-3, MTS, MEAO, MHNO, INAS)
- ☐ Hoger algemeen en voorbereidend wetenschappelijk onderwijs (bijv. HAVO, VWO, HBS, MMS, Gymnasium, Atheneum)
- ☐ Hoger beroepsonderwijs (bv. HBO, HTS, HEAO, HHMO)
- ☐ Wetenschappelijk onderwijs (WO)

België

- ☐ Geen
- ☐ Basisschool/ Lagere school regulier onderwijs
- ☐ Basisschool/ Lagere school buitengewoon onderwijs
- ☐ Lager secundair onderwijs (1ste graad SO)
- ☐ Buitengewoon secundair onderwijs
- ☐ Hoger secundair en post-secundair onderwijs (ASO, TSO, BSO)
- ☐ Professional Bachelor/Master
- ☐ Academic Master

13. Waar woont betrokkene?

- ☐ Alleen, zonder begeleiding
- ☐ Met anderen, zonder begeleiding
- ☐ Alleen, met ambulante begeleiding
- ☐ Met anderen, met ambulante begeleiding
- ☐ In een huis voor mensen met een beperking (géén 24-uurs zorg)
- ☐ In een huis voor mensen met een beperking (24 uurs zorg)

14. Kreeg betrokkene ooit één of meerdere van onderstaande diagnoses?

	**Nee**	**Ja**	**Weet niet**
Autisme spectrum stoornis (Autisme, ASS, PDD-NOS, Asperger)	☐	☐	☐
Aandachtstekort-hyperactiviteitstoornis (ADD, ADHD)	☐	☐	☐
Obsessieve-compulsieve stoornis (OCD)	☐	☐	☐
Angststoornis	☐	☐	☐
Depressieve stoornis	☐	☐	☐
Psychotische stoornis (bijv. schizofrenie)	☐	☐	☐

Andere diagnose(s), namelijk ___________________________________

 ©Van Eeghen

15. (a) Heeft betrokkene, naast de diagnose TSC, nog andere gezondheidsproblemen?

- ◻ Nee (ga naar vraag 16)
- ◻ Ja

(b) Welke gezondheidsproblemen heeft betrokkene?

- ◻ Hoge bloeddruk
- ◻ Suikerziekte (diabetes)
- ◻ Schildklieraandoening
- ◻ Kwaadaardige tumor
- ◻ Anders, namelijk ___________________________________

16. (a) Heeft betrokkene in het afgelopen jaar een ingrijpende gebeurtenis meegemaakt?

- ◻ Nee (ga naar het volgende hoofdstuk)
- ◻ Ja

(b) Wat voor ingrijpends heeft plaatsgevonden?

- ◻ Verhuizing
- ◻ Verandering van werk of dagbesteding
- ◻ Verandering in begeleiding
- ◻ Problemen met medebewoner of zorg
- ◻ Ernstige ziekte of overlijden van familie/kennis
- ◻ Een andere gebeurtenis, namelijk ___________________________________

 ©Van Eeghen

Lichamelijk functioneren

Hoe was de lichamelijke gezondheid van betrokkene in de afgelopen maand?

Zeer slecht 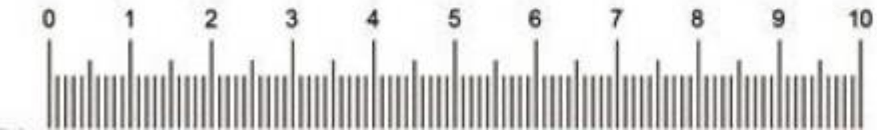 **Uitstekend**

Hieronder volgen klachten of problemen op het gebied van het lichamelijk functioneren die mensen (met of zonder TSC) kunnen ervaren. Wilt u scoren hoeveel last betrokkene heeft gehad van deze klachten gedurende de afgelopen maand? Wanneer de klachten altijd aanwezig zijn, zijn deze dus ook van toepassing op de afgelopen maand.

Geef een reactie op elke uitspraak door per rij één hokje aan te vinken.

<u>Gedurende de afgelopen maand had betrokkene last van:</u>	Heel erg	Nogal	Een beetje	Helemaal niet	Weet niet
1. moeite met slapen					
2. moeheid					
3. problemen met eten *(bijv. teveel of te weinig eten, ongewone dingen eten)*					
4. problemen met het gewicht *(bijv. onbedoeld afvallen of aankomen)*					
5. maagklachten *(bijv. maagzuur, braken, misselijkheid)*					
6. problemen met de ontlasting *(bijv. verstopping of diarree)*					

<u>Gedurende de afgelopen maand had betrokkene last van:</u>	Heel erg	Nogal	Een beetje	Helemaal niet	Weet niet
7. problemen met zien of de ogen *(bijv. niet goed kunnen zien, scheelzien)*					
8. spraak- en/of taalproblemen *(bijv. stotteren, moeilijk verstaanbaar zijn, moeite met woorden vinden)*					
9. problemen met het evenwicht *(bijv. balansproblemen, moeite met stabiel staan, lopen of zitten)*					
10. problemen van de motoriek *(bijv. onhandigheid, slechte coördinatie, stijfheid)*					

 ©Van Eeghen

Gedurende de afgelopen maand had betrokkene last van:	Heel erg	Nogal	Een beetje	Helemaal niet	Weet niet
11. huidafwijkingen					
12. ontstekingen of infecties *(bijv. griep, luchtweginfectie, blaasontsteking, aften in de mond)*					
13. epileptische aanvallen *(bijv. trekkingen, staaraanvallen)*					
14. pijn					
15. ademhalingsproblemen *(bijv. kortademigheid, piepen, hoesten)*					
16. nierproblemen					
17. spasticiteit (*verhoogde spierspanning*)					
18. vochtophoping (*bijv. enkeloedeem*)					
19. lichamelijke problemen zonder duidelijke oorzaak					

20. Had betrokkene gedurende de afgelopen maand last van bijwerkingen van de medicatie:

- □ Nee
- □ Ja, namelijk_ _

 ©Van Eeghen

<u>Geestelijk functioneren</u>

Hoe was over het algemeen de geestelijke gezondheid van betrokkene in de afgelopen maand?

Zeer slecht 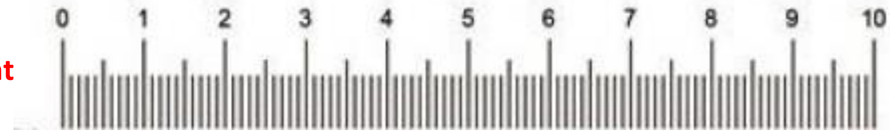 Uitstekend

Hieronder volgen klachten of problemen op het gebied van het geestelijk functioneren die mensen (met of zonder TSC) kunnen ervaren. Wilt u scoren hoeveel last betrokkene heeft gehad van deze klachten gedurende de afgelopen maand? Wanneer de klachten altijd aanwezig zijn, zijn deze dus ook van toepassing op de afgelopen maand.

Geef een reactie op elke uitspraak door per rij één hokje aan te vinken.<br><br><u>Gedurende de afgelopen maand had betrokkene:</u>	Heel erg	Nogal	Een beetje	Helemaal niet	Weet niet	Niet van toepassing
1. last van overactief of hyperactief gedrag						
2. last van rusteloos of plukkerig gedrag (*bijv. wriemelen of wiebelen*)						
3. last van impulsief gedrag (*bijv. dingen doen of zeggen zonder na te denken*)						
4. moeite met concentreren of lang de aandacht bij iets te houden *(bijv. tijdens lezen of een film kijken)*						
5. moeite met het herinneren van dingen						
6. moeite met het oriënteren in tijd en plaats (*bijv. datum kennen, weten waar hij/zij is)*						

<u>Gedurende de afgelopen maand had betrokkene:</u>	Heel erg	Nogal	Een beetje	Helemaal niet	Weet niet	Niet van toepassing
7. last van onzekerheid						
8. moeite met oogcontact maken						
9. moeite met leeftijdsgenoten om te gaan						
10. moeite met begrijpen wat iemand denkt of voelt						
11. moeite met zijn/haar eigen mogelijkheden en beperkingen in te schatten						
12. moeite met voor zichzelf opkomen *(bijv. nee zeggen*)						

 ©Van Eeghen

Gedurende de afgelopen maand had betrokkene:	Heel erg	Nogal	Een beetje	Helemaal niet	Weet niet	Niet van toepassing
13. moeite met nieuwe mensen ontmoeten						
14. moeite met veranderen van routines						
15. last van overgevoeligheid voor prikkels *(bijv. aanrakingen, fel licht, een drukke omgeving)*						
16. last van het willen herhalen van eenzelfde handeling						
17. last van koppigheid of stuursheid						

Gedurende de afgelopen maand:	Heel erg	Nogal	Een beetje	Helemaal niet	Weet niet	Niet van toepassing
18. had betrokkene stemmingswisselingen						
19. kon betrokkene moeilijk omgaan met stress						
20. raakte betrokkene snel in paniek						
21. zag of hoorde betrokkene dingen die anderen niet zagen *(bijv. hallucinaties*)						
22. kon betrokkene bepaalde gedachten moeilijk uit zijn/haar hoofd zetten						
23. had betrokkene driftbuien						
24. was betrokkene lichamelijk agressief naar anderen *(bijv. dingen gooien, schoppen slaan)*						
25. was betrokkene verbaal agressief naar anderen *(bijv. vloeken, schelden)*						
26. probeerde betrokkene zichzelf te verwonden						

Gedurende de afgelopen maand maakte betrokkene zich zorgen over:	Heel erg	Nogal	Een beetje	Helemaal niet	Weet niet	Niet van toepassing
27. epilepsie						
28. bijwerkingen van medicatie						

 ©Van Eeghen

<u>Activiteiten en participatie</u>

Kon betrokkene zijn/haar dagelijkse activiteiten ondernemen? (eventueel met hulp)

Helemaal niet 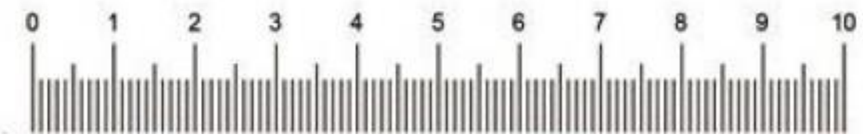 **Volledig**

Hieronder volgen activiteiten die plaatsvinden in het dagelijks leven van mensen met en zonder TSC.
Wilt u scoren hoeveel hinder betrokkene heeft ondervonden gedurende de afgelopen maand in het uitvoeren van deze activiteiten?
Geef een reactie op elke uitspraak door per rij één hokje aan te vinken.

Gedurende de afgelopen maand was betrokkene belemmerd in:	Altijd	Vaak	Soms	Nooit	Weet niet	Niet van toepassing
1. communicatie met anderen						
2. iets nieuws leren						
3. dingen onthouden						
4. twee dingen tegelijkertijd doen (multi-tasking)						
5. omgaan met mensen die hij/zij goed kent						
6. omgaan met onbekenden						
7. een band opbouwen met iemand of vrienden maken						

Gedurende de afgelopen maand was betrokkene belemmerd in:	Altijd	Vaak	Soms	Nooit	Weet niet	Niet van toepassing
8. zichzelf wassen en aankleden						
9. zelfstandig lopen						
10. sporten of aan lichaamsbeweging doen						
11. zorgen voor zijn/haar eigen gezondheid *(bijv. medicatie innemen)*						
12. zelf te kunnen bepalen wat hij/zij doet *(autonomie)*						
13. zijn/haar eigen vrije tijd plannen/indelen						
14. deelname aan dagbesteding, stage of werk						

 ©Van Eeghen

<u>Sociale steun</u>

Kreeg betrokkene in de afgelopen maand het soort steun dat hij/zij nodig had?

Helemaal niet 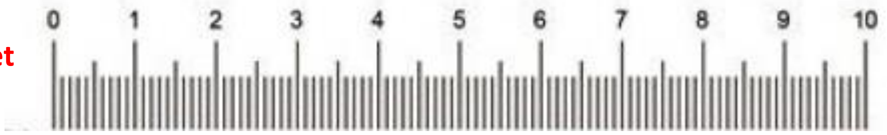 **Volledig**

In de volgende vragen wordt gevraagd naar hoe tevreden betrokkene was, of leek te zijn, met de verschillende aspecten van zijn/haar leven.

Geef een reactie op elke uitspraak door per rij één hokje aan te vinken

<u>Betrokkene was in de afgelopen maand tevreden over de steun van:</u>	Helemaal niet	Enigszins	In grote mate	Volledig	Weet niet	Niet van toepassing
1. familie/partner						
2. vrienden						
3. de patiëntenvereniging						
4. hulpverleners uit de Geestelijke Gezondheidszorg *(bijv. psychiater, psycholoog, maatschappelijk werker)*						
5. medische hulpverleners (*bijv. artsen en verpleegkundigen*)						
6. niet-medische hulpverlening (*bijv. begeleiders)*						
7. dagbesteding, werk of stage *(bijv. collega of werkgever)*						

<u>Betrokkene was in de afgelopen maand tevreden over:</u>	Helemaal niet	Enigszins	In grote mate	Volledig	Weet niet	Niet van toepassing
8. de werking van de medicijnen						
9. het huis waar hij/zij woont						
10. de beschikbaarheid van informatie over TSC						
11. zijn/haar sociale relaties						
12. zijn/haar seksuele leven						
13. zijn/haar financiële middelen						

 ©Van Eeghen

Kwaliteit van leven

De laatste vraag gaat over kwaliteit van leven.

Hoe zou betrokkene zijn kwaliteit van leven **in de afgelopen maand** inschatten?

Zeer slecht 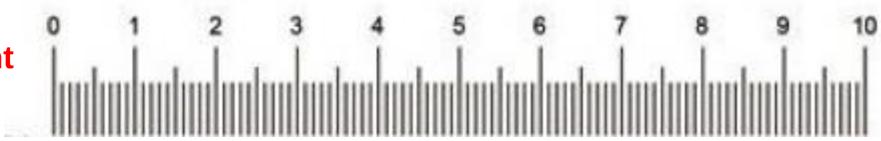

Uitstekend

Dit is het einde van de vragenlijst.

©Van Eeghen
